# Eco-Friendly Coordination Polymers with Incorporated Nitrogen-Rich Heterocyclic Ligand and Their Hybrids with Gold Nanostructures for Catalytic Conversion of Carbon Dioxide

**DOI:** 10.3390/molecules30244777

**Published:** 2025-12-15

**Authors:** Kinga Wasiluk, Gabriela Kopacka, Michał Kopyt, Piotr Kwiatkowski, Pawel W. Majewski, Elżbieta Megiel

**Affiliations:** 1 Faculty of Chemistry, University of Warsaw, Pasteur 1, 02-093 Warsaw, Poland; 2 Biological and Chemical Research Centre, University of Warsaw, Żwirki I Wigury 101, 02-089 Warsaw, Poland

**Keywords:** heterogeneous catalysis, green chemistry, CO_2_ fixation, nanoparticles

## Abstract

This paper demonstrates the successful synthesis of novel hybrid heterogeneous catalysts for the sustainable conversion of CO_2_ into cyclic organic carbonates (COCs). The nanocat-alysts have been fabricated by encapsulating pre-formed ultra-small gold nanostructures into a nascent zinc-coordination polymer (ZnCP) framework formed from two organic building blocks, 2,4-naphthalenedicarboxylic acid (1,4-NDC) and 5-amino-1H-tetrazole (5-Atz), which serves as a nitrogen-rich ligand. Applying the fabricated catalysts in the synthesis of COCs yields high yields (up to 97%) and high selectivity (up to 100%), with exceptionally high turnover frequencies (TOFs) (up to 408 h^−1^). The catalytic process can be carried out under mild conditions (80 °C, 1.5 MPa CO_2_) and without the use of solvents. Nitrogen-rich ligand molecules in the structure of ZnCPs enhance catalytic performance thanks to additional nucleophilic centres, which are effective in the epoxides’ ring-opening process. The hybrid catalysts with encapsulated gold nanostructures, which modify the liquid–gas interface between epoxide and CO_2_, give significantly higher yields and TOFs for less active epoxides. The designed hybrid nanocatalysts exhibit superior stability under the studied reaction conditions and can be reused without loss of activity. The developed coordination polymers are constructed from green components, and green chemistry principles are applied to prepare these catalytic materials.

## 1. Introduction

Solid-state heterogeneous catalysts are highly desirable in sustainable chemistry, as their application in industrial processes reduces waste generation and yields higher turnover rates than their soluble counterparts [1,2,3,4]. In the most recent two decades, intensive efforts have been dedicated to designing coordination polymers (CPs) as novel heterogeneous nanocatalysts for green chemistry [5,6,7,8]. CPs are a diverse group of advanced materials with one-dimensional or higher-ordered arrangements formed primarily thanks to coordination bonds between metal ions or their clusters and rigid organic building blocks. Porous coordination polymers are usually distinguished and named as metal–organic frameworks (MOFs) [5,9]. Three main factors should be considered in designing CPs for sustainable catalytic processes. First, metal ions in their structures must be environmentally benign; second, the stability of these materials must enable their use as recyclable catalysts; finally, their synthesis must be performed without hazardous reagents and under mild conditions. In this aspect, zinc-based coordination polymers (ZnCPs) definitely stand out. Zn(II) ions can serve as effective Lewis acid sites for coordinating with substrate molecules, exhibiting good catalytic activity in many chemical processes [10]. At the same time, this element is biocompatible and environmentally safe [11]. The intriguing photoluminescence behaviour of ZnCPs has also been intensively explored over the last decade due to their potential applications as optical sensors, molecule-scale photodevices, optical logic gates, and others [12].

Climate change mitigation is one of the most pressing challenges of modern times and a crucial sustainable development goal [13]. This aim can be realised, among other things, by reducing anthropogenic CO_2_ emissions through its capture from flue gases and utilisation; this strategy is known as Carbon Capture and Utilisation (CCU) [14]. Thus, new materials that enable effective CO_2_ capture and simultaneously activate this kinetically and thermodynamically stable compound as a heterogeneous catalyst are highly desirable. Importantly, CO_2_ can be used as a C1 Synthon to synthesise many organic compounds [15,16,17,18,19].

Fixation of CO_2_ using epoxides, leading to cyclic organic carbonates (COCs), also known as the cycloaddition of CO_2_, is recognised as one of the most effective methods for CO_2_ utilisation. First, it runs with 100% atom efficiency, meaning all substrates’ atoms are built into the product molecule; furthermore, the fabricated products, the COCs, have many applications; among others, they can be used as electrolytes in lithium-ion batteries, polar high-boiling-point solvents, components of cosmetics, synthons or intermediates in synthesis carbamates, 1,2-diols, monomers for the production of biodegradable polymers and many others [20,21,22,23,24,25,26,27,28].

ZnCPs are perfect candidates for CO_2_ adsorption and heterogeneous catalysts for their chemical transformation; thus, they have been intensively explored in recent years. Zinc ions in the CPs network are strong Lewis-acidic centres that activate the epoxide ring through the oxygen atom, and nucleophilic anions in their structures enable the ring to open and the insertion of the CO_2_ molecule [21,29,30]. The amphoteric character, high affinity towards CO_2_, and porous morphology of these materials enhance CO_2_ adsorption [10,31,32]. CO_2_ capture capability can be additionally facilitated by properly designed ligands in the structure of CPs. Tran et al. used 4,4′-(ethyne-1,2-diyl)bis(2-oxidobenzoate) as a building block to obtain M-MOFs (M = Mg, Ni, Co, Zn, Cu, Fe) with high porosity, labelled as MOF-184. Among the synthesised, Zn-MOF-184 showed the best catalytic activity for the cycloaddition of CO_2_ to epoxides. Interestingly, the performance of these CPs correlates very well with the strongest Lewis acidity of the Zn cations among those studied and the ligand’s high basicity, as determined using vapour adsorption and ATR-FTIR spectroscopy [33]. Agarwal and *co*-workers used two organic building blocks for the synthesis of Zn-MOFs: the classical ligands tricarboxylic acid derivatives of benzene and 1,3,5-tri(1H-benzo[d]imidazol-1-yl)benzene, both of which contain nitrogen atoms that serve as alkaline centres [34]. This approach enabled them to obtain highly efficient heterogeneous catalysts for coupling epoxides with CO_2_ under mild conditions. Very recently, the synthesis and catalytic activity of CPs obtained using two ligands, (4-carboxyphenyl)-5-mercapto-1H-tetrazole and 4,4′-bipyridine, were reported [35]. The fabricated material, thanks to its dual character caused by the presence of Lewis acidic and alkaline sites, yielded high amounts of epichlorohydrin carbonate (98%) and styrene carbonate (82%) after 24 h of reaction time at 70 °C under atmospheric CO_2_ pressure [35]. Interesting results were also obtained for carboxyl-containing and amine-rich Zn-MOFs fabricated using the ligands 2,5-thiophene-dicarboxylic acid and melamine with triethylamine as an additional deprotonation agent. The synthesised materials with hierarchical pores exhibit a high CO_2_ adsorption capacity and good catalytic activity in the fixation of CO_2_ to epoxides [36]. Azam and *co*-workers recently reported that transition-metal-based coordination polymers (CPs) synthesised with the primary ligand 4-aminonaphthalene-2,6-dicarboxylic acid and several aromatic amines as auxiliary ligands exhibit highly ordered crystal structures and exceptionally high stability [37]. The catalytic activity of these materials in the chemical conversion of CO_2_ to cyclic carbonates and their adsorption capacity were not tested. On the other hand, it was reported that CO_2_ preferential capture by ZnCPs was achieved with 4-amino-1,2,4-triazole and 5-aminotetrazole as the sole ligands [38,39]. Also, very recently, a synergistic effect in nitrogen-coordinated zinc for CO_2_ cycloaddition [40]. This indicates that incorporating nitrogen-rich ligands in the CP structure should enhance CO_2_ transformation inside the framework. Simultaneously, additional ligands with rigid structures, such as dicarboxylic aromatic molecules, are expected to provide better stability and higher porosity in the obtained coordination polymers. To date, no such advanced framework has been reported that combines amino-derivatives of tetrazole containing several nitrogen atoms in the molecule’s ring, some of which have lone pairs of electrons available to accept protons with a rigid linker in one framework.

The hybrid materials of metal nanoparticles (MNPs) and CPs represent a novel, unexplored field in heterogeneous catalysis and have attracted significant attention. Combining MNPs with CPs enhances the catalytic activity of both components when used separately, resulting in a synergistic effect [41]. Notably, the encapsulation of MNPs within the CPs’ structure protects against aggregation even under harsh conditions [42]. Hybrid materials obtained via immobilisation of gold nanoparticles (AuNPs) in CP structure are up-and-coming because of the unique physicochemical properties of these nanostructures and their excellent catalytic activity in many reactions [43,44,45,46,47,48,49]. Recently, we demonstrated that the presence of AuNPs in aluminium-based MOF catalysts facilitates the coupling of CO_2_ with propylene oxide, yielding a two-fold higher yield compared to analogous catalysts without these nanostructures [45].

Herein, we report the synthesis of novel ZnCPs using two organic building blocks, naphthalene-1,4-dicarboxylic acid and 5-aminotetrazole, and their hybrids containing gold nanostructures. The successful employment of the fabricated materials as recyclable catalysts for sustainable CO_2_ fixation is presented. These designed hybrid catalysts have been fully characterised using XPS, ^1^H NMR, ICP-MS and elemental analysis, SEM and TEM imaging, SEM-EDS spectroscopy, CO_2_ and N_2_ adsorption measurements (BET), PXRD and TGAs.

## 2. Results and Discussion

**Scheme 1:**
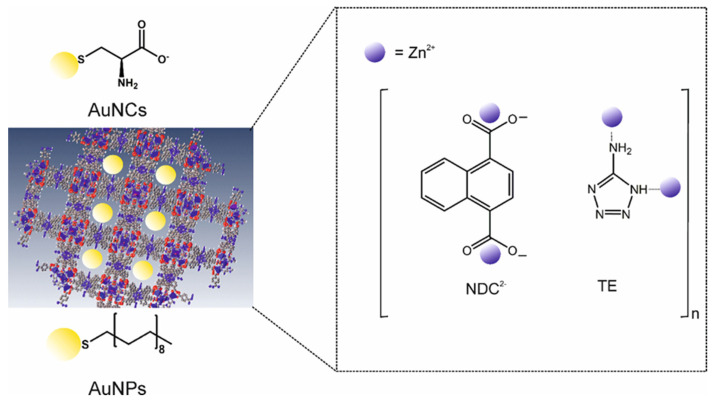
Schematic representation of the structure of the fabricated ZnCPs hybrids with encapsulated gold nanostructures.

Zinc-based coordination polymers (ZnCPs) were synthesised using two organic building blocks, 1,4-naphthalenedicarboxylic acid (1,4-NDC) and 5-amino-1H-tetrazole (5-Atz), in a self-assembled structure. We employed two methods for synthesis: solvothermal and a more environmentally friendly diffusion-controlled method [50]. The hybrid materials of these polymers with gold nanoparticles (AuNPs) and gold nanoclusters (AuNCs) were fabricated using the “bottle around the ship” strategy [41,42]. Three ligand species build the framework of these materials: deprotonated 1,4-naphthalene dicarboxylic ions (NDC^2−^), 5-amino-tetrazole (TE) and protonated 5-amino-tetrazole (TE^+^). Pores of the fabricated coordinated polymers are partially occupied by gold nanostructures (Scheme 1).

### 2.1. Structure Studies of Nanocatalysts

Table 1 presents the determined composition of the fabricated nanocatalysts. From the nitrogen and carbon contents, the molar ratio between 1,4-NDC and 5-Atz in the structures was approximately 1:1, where part of the amine ligand molecules were protonated (labelled as TE^+^ in Table 1). In hybrid materials, the content of gold is below 1% in Zn_5TE_AuNPs@NDC and 2.5% in the hybrid material obtained using gold nanoclusters (Zn_5TE_AuNCs@NDC).

XPS analysis was performed for the hybrid material of ZnCPs with AuNCs. The general XPS spectrum is presented in the Supplementary Materials (Figure S1).

**Figure 1:**
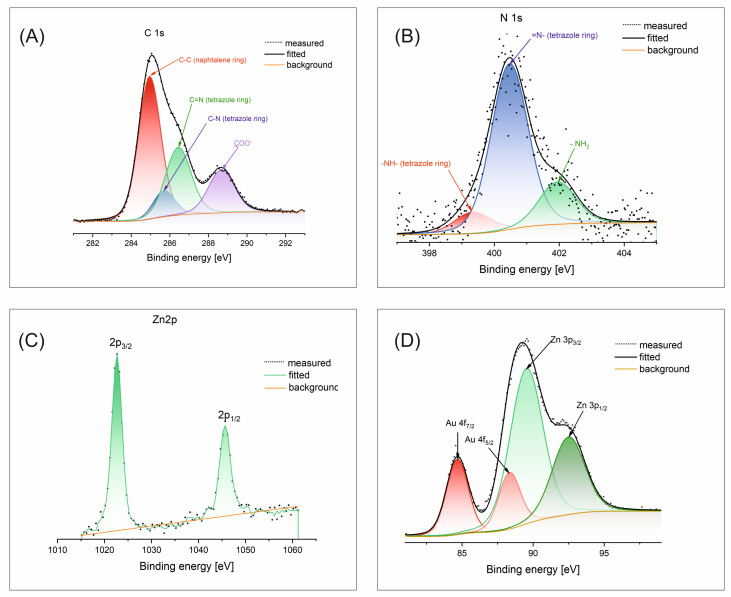
The selected HR-XPS spectra for Zn_5TE_AuNCs@NDC.

Figure 1 displays high-resolution XPS spectra of C 1s, N 1s, Zn 2p and Au 4f orbital regions. In the C 1s spectrum (Figure 1A), four peaks can be designated at 285 and 288.7 eV, corresponding to C-C and COO^−^ bonds in the naphthalene ring and these positions are consistent with literature data [51], whereas 286.4, 285.7 eV can be attributed to -C=N and -C-N bonds in tetrazole ring, respectively, very similar to positions determined by Bikas et al. [52] for the tetrazole Zn-coordination complex. The carbon content, as determined by XPS analysis, is 36.4% (Table 2), which agrees with that obtained from elemental analysis (35.4%).

The N 1s spectrum (Figure 1B) yields three peaks from deconvolution at 399.3, 400.5, and 402 eV, which can be attributed to -NH-, =N-, and -NH_2_ bonds, respectively. Similarly located peaks were reported for the Zn-coordination complex with 5-Atz [52].

Figure 1C displays the XPS spectrum for Zn 2p orbitals. The Zn 2p_3/2_ peak is centred at 1022.7 eV, which is 0.9 eV higher than that observed for Zn in ionic form, for example, in ZnO. It can be explained by interactions between Zn and the tetrazole moiety in the polymer framework, which decrease the valence-electron density of Zn and thereby increase the binding energy of its inner orbitals. A similar phenomenon was reported for Zn-doped MFI-type zeolite frameworks, where interactions between Zn and more electronegative Si atoms were observed [53]. The Zn 2p_1/2_ is located at 1047.3 eV, which is 2–3 eV higher than typical for Zn^2+^, which can be explained by the coordination of hydroxyl groups in the structure, as recently identified by Cempura et al. [54]. The determined zinc content agrees with the ICP-MS data.

The Au 4f–Zn 3p HR spectra can be deconvoluted into four peaks (Figure 1D), namely Au 4f_7/2_ (84.7 eV), Au 4f_5/2_ (88.3 eV), Zn 3p_3/2_ (89.6 eV) and Zn 3p_1/2_ (92.5 eV). At the same time, the position of these peaks corresponds to those observed in the case of Zn-Au alloys and sulphide-coated metallic gold, indicating charge transfer between Au atoms and Zn^2+^ ions when they are close neighbours [55,56,57]. Gold content from XPS agrees with the result from SEM-EDS.

High-resolution spectra of S2p_3/2_ and O 1s orbitals are shown in Supplementary Materials (Figure S2A). The broad S 2p spectrum was deconvoluted into four peaks corresponding to the 2p_3/2_ and 2p_1/2_ orbitals in different environments. The peaks centred at 163.1 eV (2 p_3/2_) and 164.3 eV (2 p_1/2_), split by 1.2 eV, are in perfect accordance with literature data for Au-S bonds formed by dialkyl disulfides, dinitroxyl disulfides and alkyl thiols on gold clusters with diameters from 1.5 nm to 5.2 nm [47,49,58]. In the studied sample, Au-S bonds form between the gold nanoclusters’ surface and the sulphur atoms in L-cysteine molecules. The peaks at higher energies, 169.0 eV and 170.3 eV, most likely indicate the presence of polysulfides formed from L-cysteine during the synthesis of clusters, which were reported as intermediate products [59].

The HR spectrum of O 1s (Figure S2B) can be deconvoluted into two peaks at 532.1 eV corresponding to the –C=O and –OH^-^ moieties and centred at 533.3 eV attributed to –C-O bonds from deprotonated carboxylic groups [60] in the 1,4-NDC building block.

**Figure 2:**
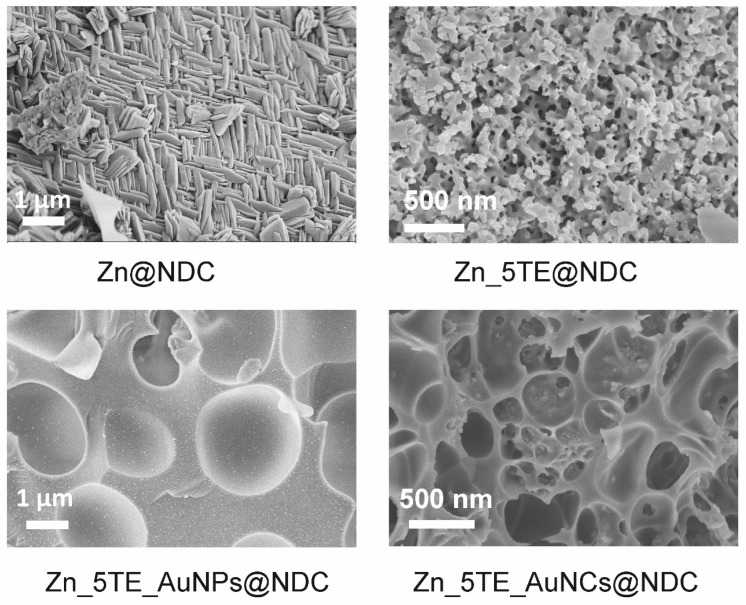
Selected SEM images of the fabricated nanocatalysts.

Figure 2 displays the selected SEM images for ZnCPs with only 1,4-NDC (Zn@NDC) and built with 5-Atz (Zn_5TE@NDC). The morphology differs, and higher porosity is evident in the last mixed framework.

**Figure 3:**
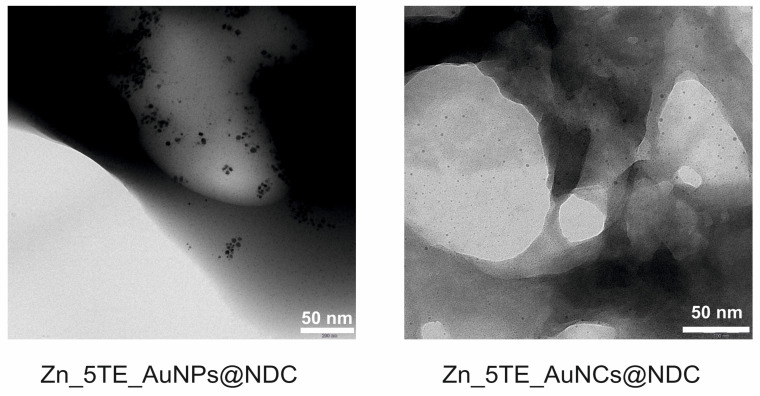
TEM micrographs of hybrid catalytic materials.

The hybrid materials obtained using gold nanoparticles (AuNPs) and gold nanoclusters (AuNCs) via the “bottle around the ship” strategy differ in morphology, forming spherical particles with internal surfaces coated by nanostructures. It is also visible in TEM images (Figure 3). Furthermore, ultra-small nanoparticles are observed to be embedded inside the pores of the hybrid materials. Additional SEM and TEM images with varying magnifications are presented in the Supplementary Materials (Figures S3 and S4).

### 2.2. Physicochemical Characterisation of Nanocatalysts

**Figure 4:**
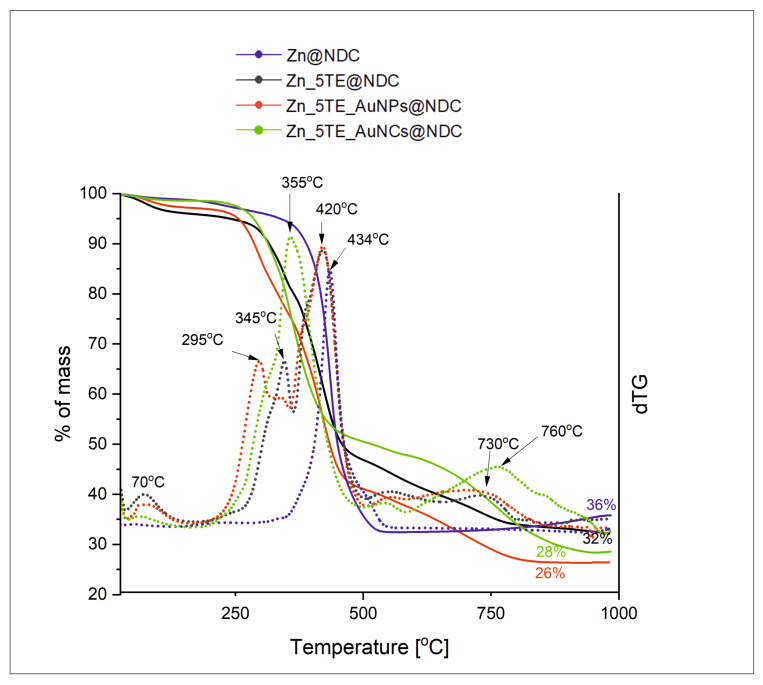
TG curves recorded for the fabricated nanocatalysts under a nitrogen atmosphere (solid lines) and their derivative curves (dotted lines), where the TG and corresponding dTG curves are in the same colour.

The results of the thermogravimetric analyses (TGA) demonstrated the high thermal stability of the fabricated nanocatalysts. Figure 4 illustrates TGA curves recorded for CPs obtained solely with 1,4-NDC (Zn@NDC), built of two organic blocks (Zn_5TE@NDC) and its hybrids with gold nanoparticles (Zn_5TE_AuNPs@NDC) and nanoclusters (Zn_5TE_AuNCs@NDC) under a nitrogen atmosphere during the heating up to 1000 °C. Also, the corresponding derivatives of these curves are presented as dTG curves. In the case of Zn@NDC, thermal decomposition is a one-step process with a maximum temperature of 434 °C, yielding a solid residue of 36%. The first weight loss for the other studied materials, up to 70 °C, corresponds to the removal of guest water molecules (approximately 10%). When an additional ligand is present in the structure of Zn_5TE@NDC, the process consists of two stages: the first step, at 345 °C, corresponds to its decomposition, and the second peak is similarly located for Zn@NDC. The decay of Zn_5TE_AuNPs@NDC begins at lower temperatures, likely due to 1-octadecanethiol, which acts as a stabilising agent for AuNPs and degrades as the first organic component [61]. Afterwards, the 5-Atz and 1,4-NDC moieties decompose, resulting in around 60% weight loss. Slight weight loss is observed above 500 °C, and the solid residue after decomposition is slightly higher for Zn_5TE@NDC (32%) than for its composite with AuNPs (26%). This can be attributed to the higher organic fraction in Zn_5TE_AuNPs@NDC, which is due to the nanoparticles’ stabilising layer. A similar content of solid residue was determined for Zn_5TE_AuNCs@NDC (28%). However, for this material, a maximum of thermal decomposition begins at a higher temperature than for Zn_5TE_AuNPs@NDC, and a broad peak of the dTG curve with a maximum at 355 °C corresponds to the decay of 5-Atz, 1,4-NDC and cysteine together; the decay of a small fraction of the PEI layer can explain the subsequent broad weight loss [62].

**Figure 5:**
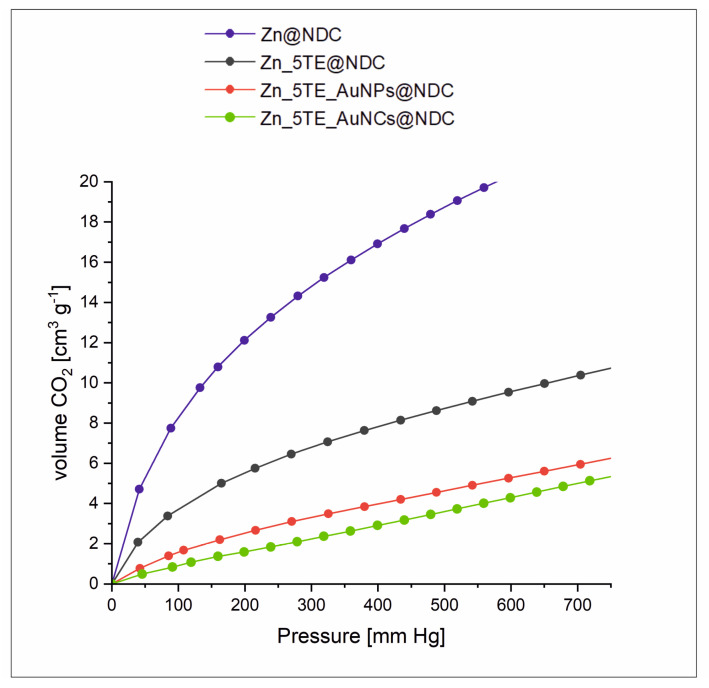
CO_2_ adsorption isotherms recorded at 273 K for the fabricated catalysts.

Isotherms of N_2_ adsorption for Zn5TE@NDC and its composite with AuNPs, recorded at 77 K, are presented in Figure S5. According to Brunauer classification, they are reversible Type-1. Regarding the studied catalytic applications of CO_2_, its adsorption at 273 K is crucial; we utilise isotherms measured for this gas under these conditions to evaluate its porosity (Figure 5). The Brunauer–Emmett–Teller (BET) surface areas and pore volumes were determined for all the fabricated nanomaterials (Table 3). The highest BET surface area (S_0_) is observed for the material obtained using only 1,4-NDC as a ligand, at 146 m^2^ g^−1^; a two-times lower value was determined for ZnCPs with 5-Atz as the second ligand. The determined surface area for Zn_5TE@NDC is very close to that obtained by Zhao et al. [63] for Zn-MOF. Significantly smaller S_0_ values were determined for hybrid materials. These changes in porosity can be explained by the fact that gold nanostructures may occupy some of the pores formed in the network. The sizes of pores in the fabricated materials are below 2 nm; thus, all fabricated nanocatalysts can be classified as microporous. For the DFT pore size distribution, see Figure S6.

Figure S7 shows absorbance spectra recorded for gold nanoparticles (AuNPs) and nanoclusters (AuNCs) used to fabricate nanocatalysts in their toluene and water solutions, respectively. The surface plasmon resonance (SPR) band is visible at 520 nm in the spectrum of gold nanoparticles (AuNPs). The spectrum of AuNCs does not exhibit a visible SPR band, confirming that their diameter is approximately 1–2 nm [66,67]. Instead of the SPR band, strong absorbance is visible at 380 nm and 870 nm. Such localised bands indicate that the synthesised clusters are neutral, with a general formula of Au_25_(SR)_18_ [68]. In the spectrum of AuNPs, bands at 380 nm and 870 nm are also observed, in addition to the SPR band at 520 nm, indicating the presence of a fraction of smaller nanoparticles. These conclusions are consistent with insights from TEM analyses (Figure S8).

**Figure 6:**
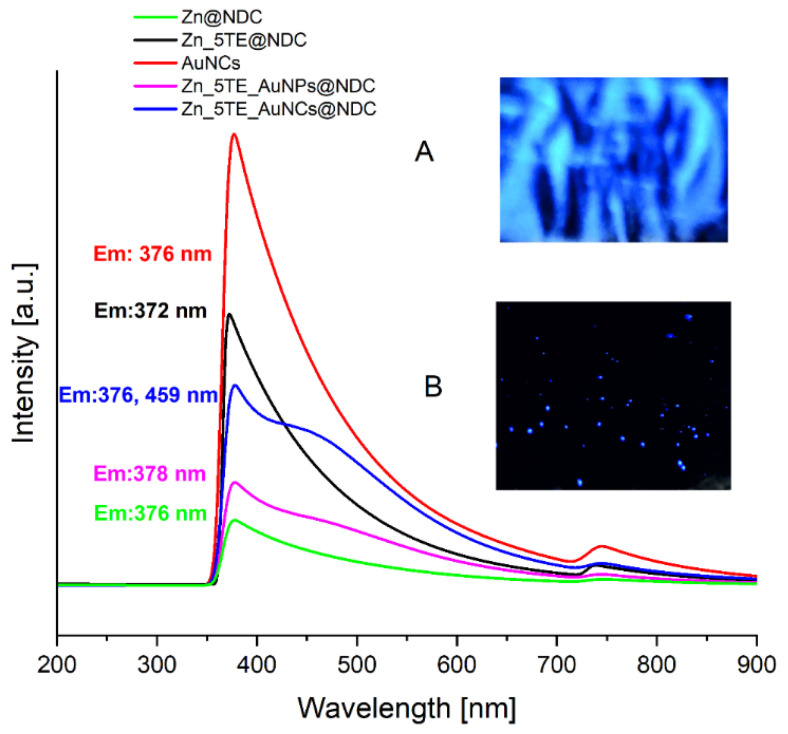
Fluorescence spectra of AuNCs in water solution and Zn-based CPs in a solid state (λ_ex_ = 365 nm). Inset (**A**) is a photo of the fluorescence of Zn_5TE_AuNCs@NDC in powder form. (**B**) is the fluorescence of single crystals of this hybrid material (Exc. 365 nm).

Strong blue fluorescence is observed for the fabricated ZnCPs, AuNCs and their hybrids. Figure 6 displays the fluorescence spectra of all fabricated materials under 365 nm excitation, along with photos of the fluorescence of the Zn_5TE_AuNCs@NDC composite material in the solid state under 365 nm excitation. The weakest fluorescence was observed for Zn@NDC, with a maximum emission at 376 nm. The 5-Atz ligand in Zn_5TE@NDC shows a stronger emission, slightly shifted to a shorter wavelength. For the hybrid materials with gold nanostructures, a strong band at 376 nm is observed, analogous to that in the solution of the fabricated nanoclusters. Additionally, a weak band at 459 nm is present, which is less visible in Zn_5TE_AuNPs@NDC than in Zn_5TE_AuNCs@NDC.

**Figure 7:**
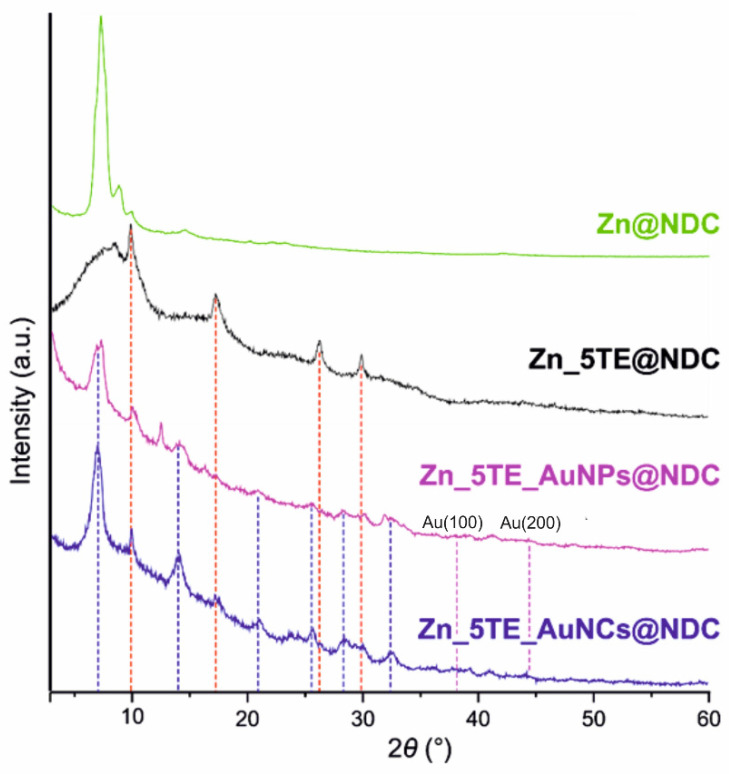
PXRD patterns of the obtained nanomaterials. Vertical red dashed lines indicate reflexes found in all CP materials, and blue dashed lines denote reflexes common to Au-containing CPs. Pink dashed lines indicate the expected positions of crystalline gold reflexes (100) and (200). Vertical lines serve only as a guide to the eye.

Powder X-ray Diffraction (PXRD) patterns of the CPs are shown in Figure 7. The diffraction pattern of Zn@NDC closely resembles that of the octanuclear zinc(II) carboxylate framework reported by Meng et al. [69]. However, weaker reflections are observed at higher diffraction angles. The presence of the 5-Atz ligand induces crystallisation of the Zn_5TE@NDC (black curve) in a crystallographic system different from that of the CPs formed only with 1,4-NDC (green curve).

In Zn_5TE@NDC and in gold hybrids, at least two phases are observed. The broader, less intense diffraction peaks in Zn_5TE@NDC indicate the presence of an amorphous or poorly crystalline phase that appears to further develop upon the incorporation of gold, whether as nanoparticles or nanoclusters.

This suggests that the presence of gold nanostructures promotes faster formation or maturation of the target crystalline phase. The diffractogram of the CP containing gold nanoclusters closely resembles that of the CP with AuNPs. Notably, no diffraction peaks corresponding to crystalline gold are observed in samples containing AuNPs or AuNCs. The expected positions of the characteristic Au(100) and Au(200) reflections are marked in Figure 7 with vertical dashed pink lines. Although the two gold-containing samples are similar (blue vertical dashed lines), the one containing AuNPs exhibits additional reflexes associated with a minor unidentified phase, which does not correspond to crystalline gold. We note that the absence of crystalline Au reflexes is expected due to the very small size of both AuNPs (<4 nm) and AuNCs (<2 nm) and their low content (0.5 and 2.5 wt%, respectively).

Importantly, none of the samples, including Zn_5TE@NDC and those containing gold nanostructures, exhibit diffraction peaks characteristic of 1,4-NDC or 5-Atz (Figure S18), which proves that ligands not connected in polymer framework are not present in the obtained structures and do not occupy micropores as free ligands.

### 2.3. Catalytic Assays

**Scheme 2:**
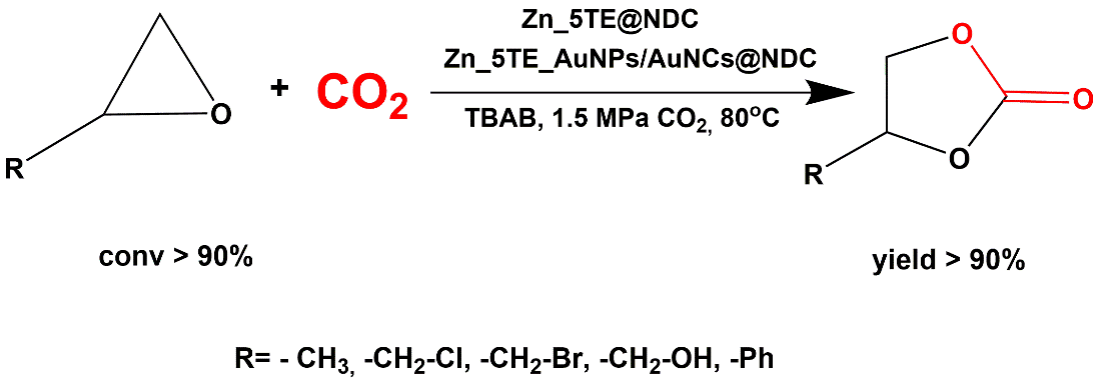
General scheme of the studied catalytic reactions.

All the synthesised materials have been tested as heterogeneous catalysts in the fixation of CO_2_ with model epoxides using tetrabutylammonium bromide (TBAB) as a co-catalyst (Scheme 2).

Table 4 presents the results of catalytic assays conducted for five model epoxides, which differ in their activity. All reactions were carried out without solvent on a scale of 14–20 mmol of epoxide (80 °C, 1.5 MPa CO_2_). For propylene oxide, we compared all the types of catalysts. As this epoxide belongs to the less active class, 24 h was needed to obtain satisfactory yields. The presence of 5-Atz in the catalyst structure significantly improves its performance in CO_2_ fixation to propylene oxide, resulting in a 20% increase in yield.

On the other hand, the encapsulation of gold nanostructures enables reaction yields above 80%. For Zn_5TE_AuNCs@NDC, the results are the best; propylene carbonate was obtained with a yield of 90% and a TON parameter above 1000. It is worth noting that the method for synthesising this commercially attractive compound is both sustainable in terms of CO_2_ management and facilitates simple product separation.

Thanks to high selectivity and high epoxide conversion, the only component that needs to be separated after the reaction is the co-catalyst, which can be adsorbed onto a suitable sorbent, allowing for a high-purity product to be obtained without additional purification steps.

Since Zn_5TE@NDC’s performance was better than Zn@NDC’s, we tested only catalysts based on this polymer in subsequent reactions. For the most active epoxides, such as epibromohydrin and glycidol, the developed catalysts yielded excellent results in very short reaction times (3–4 h). When the Zn_5TE_AuNCs@NDC catalyst was used, 100% glycidol conversion was observed after 4 h. Meanwhile, the application of this catalyst for the fixation of CO_2_ with epibromohydrin yielded 96% carbonate within 3h, with selectivity approaching 100%. Additionally, high conversions of epichlorohydrin and styrene oxide were achieved using the developed catalysts under the studied conditions, although longer reaction times were required (20 h for epichlorohydrin and 24 h for styrene oxide). It is worth emphasising that the brilliant high selectivity (close to 100%) of these epoxides towards the proper carbonates was achieved.

We optimised reaction conditions, including time, temperature, and catalyst and co-catalyst amounts, for reactions with epibromohydrin (EPI-Br).

**Figure 8:**
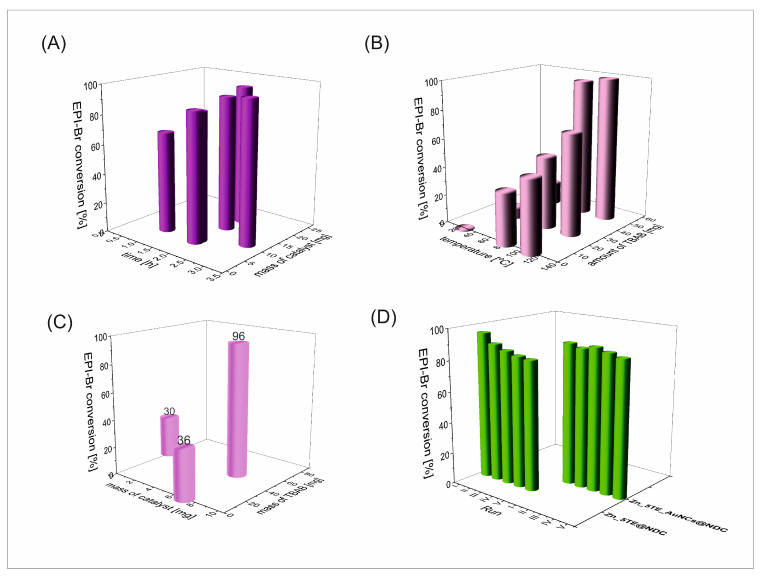
The influence of reaction conditions with Zn_5TE_AuNCs@NDC as a catalyst on the conversion of epibromohydrin (EPI-Br): (**A**–**C**); conversion of EPI-Br in five subsequent cycles (**D**) (2 h, 80 °C, 1.5 MPa CO_2_, 15 mg of catalysts in the first cycle, 50 mg of TBAB, 14 mmol of EPI-Br).

Figure 8A shows the effect of Zn_5TE_AuNCs@NDC mass on the EPI-Br conversion in the range of 5 to 20 mg at 80 °C. After 2 h of reaction, the conversion of EPI-Br across the entire range was above 80%, with the highest value observed for 20 mg of catalyst. After 3 h, a 95% conversion was achieved with 7 mg of catalyst; this mass was then used in subsequent catalytic assays. At a temperature of 25 °C, the reaction of cycloaddition of CO_2_ to EPI-Br with this mass of catalyst runs very slowly, and after 3 h, conversion is 10% with 7 mg of Zn_5TE_AuNCs@NDC and 25 mg of TBAB, 20% when, at this temperature, the mass of TBAB was increased to 50 mg (Figure 8B). As optimal, we choose a temperature of 80 °C and 50 mg of TBAB (conversion above 95%). Figure 8C illustrates the synergistic effect of the catalyst and *co*-catalyst. When only 50 mg of TBAB is used in the reaction with EPI-Br (without the catalyst), a 30% conversion is obtained after 3 h at 80 °C. A higher EPI-Br conversion was observed when the reaction was performed without TBAB but with 7 mg of catalyst. When the mixture of 7 mg of Zn_5TE_AuNCs@NDC and 50 mg of TBAB was applied, the conversion after 3 h at 80 °C was 96%. Notably, the yields of the reactions were very close, indicating high selectivity.

The recyclability of the fabricated nanocatalysts was also tested for reactions with EPI-Br as a model substrate. Figure 8D displays the results of experiments conducted in five consecutive runs, showing the conversion of this epoxide using Zn_5TE@NDC and Zn_5TE_AuNCs@NDC as catalysts. As shown, both catalysts can be reused five times without significant loss of activity; for the last catalyst, slightly higher and stable conversion values were obtained in consecutive runs. Additionally, the yields of the corresponding carbonate in five subsequent runs were high, affording the product with a remarkable 75% yield after the fifth run for both tested catalysts (Figure S9). SEM-EDS measurements showed that the composition of the catalyst after the use in five subsequent catalytic cycles is very similar to that determined after its synthesis (see Table S2 in the Supplementary Materials).

Linear relationships between ln[EPI-Br] and reaction time (Figure S10) indicate the first-order kinetics of the studied reactions using Zn5_TE@NDC and Zn5_TE_AuNCs@NDC as catalysts. The kinetic data for Zn_5TE@NDC and its hybrid with AuNCs for this active epoxide are very similar, with a slightly higher rate constant for Zn_5TE@NDC. It suggests that gold has no significant influence on the reaction rate in catalysts. However, it is vital to note that for less active epoxides, such as propylene oxide and styrene oxide, significantly higher conversions and yields (TON > 1000) were obtained when catalysts containing gold nanostructures were employed (Table 4). The synergistic effect between Zn and Au can presumably explain the superior catalytic performance of these hybrid catalysts. Jiang and *co*-workers observed a similar phenomenon in the Zeolitic Imidazole Framework (ZIF-8) containing Au and Zn ions during the coupling of CO_2_ to propylene oxide [70]. Heterobimetallic Au/Zn-MOFs were obtained using an ion exchange process. The Kirkendall effect on the ZIF-8 structure, constructed from Zn^2+^ ions linked by 2-methylimidazole, resulted in hollow nanostructures, enabling the production of propylene carbonate with a yield exceeding 90% and TOF of 11.7 h^−1^ under 3 MPa CO_2_. In contrast to our hybrid catalysts, the gold component was incorporated in an ionic form (Au^3+^) coordinated to the linker’s nitrogen atoms. Our catalytic material enables us to observe this synergistic effect between these elements for the metallic form of Au at a 100 times lower concentration. We obtained the product with a similar yield using a significantly lower number of catalysts, achieving a 9times-higher TOF for our system with Zn_5TE_AuNCs@NDC. Table S1 compares Zn_5TE_AuNCs@NDC with other reported CP systems for styrene carbonate synthesis. Our system enables higher conversion and yield under significantly milder conditions, using a recyclable catalyst that requires less energy. Thus, the proposed approach is more sustainable compared to what has been published to date.

## 3. Materials and Methods

**Materials:** Zn(NO_3_)_2_•6H_2_O, ACS reagent, ≥98% (Merck, Kenilworth, NJ, USA), 1,4-naphtalene dicarboxylic acid (1,4-NDC), 98% (AmBeed, Arlington Heights, IL, USA), HAuCl_4_•3H_2_O, ACS reagent, ≥49.0% Au basis (Merck), 1-octadecanethiol ≥ 98% (Merck), tetraoctylammonium bromide [CH_3_(CH_2_)_7_]_4_NBr TOAB, 98% (Merck), tetrabuthylammonium bromide ([CH_3_(CH_2_)_3_]_4_NBr) TBAB, 98% (Merck), NaBH_4_, powder ≥ 98% (Sigma Aldich, St. Louis, MO, USA), glycidol, 96% (Thermo Scientific, Waltham, MA, USA), Epichlorohydrine (EPI), 99% (Thermo Scientific), Epibromohydrine (EPI-Br), 99% (Thermo Scientific), Styrene oxide (St-O) ≥ 97% (Thermo Scientific), (+/−) Propylene oxide (Pr-O), ≥99% (Thermo Scientific), dimethylformamide DMF (≥95%, Thermo Scientific), dichloromethane (DCM, Thermo Scientific) ≥ 95%, ethyl acetate (≥95%, Thermo Scientific Chemicals), toluene (≥95%, Thermo Scientific Chemicals), ethanol anhydrous (≥99%, Thermo Scientific Chemicals), 5-amino-1H-tetrazole (5-Atz), 95% (Angene, Hong Kong, China), anisole (anhydrous, 99.7%), (Thermo Scientific Chemicals), propylene carbonate, anhydrous 99.7%, Merck, 4-(Hydroxymethyl)-1,3-dioxolan-2-one, ≥98%, Merck, methanol anhydrous, 99% (Merck), PEI (hyperbranched M_w_ = 800 Da by GPC, Sigma-Aldrich), L-Cysteine (97%, Merck), Triethylamine, anhydrous, 99% (Thermos Scientific Chemicals), Ethanol absolute for analysis, 99.9% (Merck), CO_2_ (compressed, Air Products, Allentown, PA, USA).

**Synthesis of Zn@NDC/Zn_5TE@NDC**

I. **Solvothermal method:** 238 mg of Zn(NO_3_)_2_•6H_2_O (0.8 mmol), 34 mg (0.4 mmol) of 5-Atz (in the case of Zn_5TE@NDC), and 173 mg of 1,4-NDC (0.8 mmol) were added to 10 mL of DMF. The solution was stirred for 10 min using a magnetic stirrer; it was transparent after this. Next, the solution was transferred to a 35-mL Synthware pressure vessel, sealed with an O-ring PTFE screw cap and heated in an oven for 72 h at 110 °C. In the case of Zn@NDC, only Zn(NO_3_)_3_•6H_2_O and 1,4-NDC were added in identical amounts, but without the amine. Afterwards, the precipitated crystals were filtered, thoroughly rinsed with methanol and dried in air. The dried crystals were shaken with anhydrous methanol and exchanged three times (30 mL per step) using a lab shaker for 24 h at 200 rpm. After this time, the mixture was centrifuged at 1500 rpm for 15 min, and the supernatant was discarded. The solid was then dried in a vacuum oven for 4 h at 60 °C (10 mbar) and subsequently activated by heating in the oven under normal pressure at 165 °C for 24 h. Zn@NDC and Zn_5TE@NDC were obtained as beige-coloured powders (123 mg and 80.4 mg, respectively). Yields: Zn@NDC 71%, Zn_5TE@NDC 39%.

II. **Diffusion method:** 238 mg of Zn(NO_3_)_2_•6H_2_O (0.8 mmol), 34 mg (0.4 mmol) of 5-Atz (in the case of Zn_5TE@NDC), and 173 mg of 1,4-NDC (0.8 mmol) were placed in a vial and dissolved in 20 mL of anhydrous ethanol. The vial was covered with plastic foil, and four holes were made in this foil using a needle. In the second vial, 5 mL of triethylamine (TEA) was dissolved in 15 mL of anhydrous ethanol. The vial was then covered with plastic foil, and four holes were made in this foil using a needle. The vials were placed in a beaker, the vessel was tightly closed, and the mixture was left for 48 h. After this time, the precipitated solid in the mother solution was centrifuged, thoroughly rinsed with methanol and dried in air. The dried crystals were shaken with anhydrous methanol and exchanged three times (30 mL per step) using a lab shaker for 24 h at 200 rpm. After this time, the mixture was centrifuged at 1500 rpm for 15 min, and the supernatant was discarded. The solid was then dried in a vacuum oven for 4 h at 60 °C (10 mbar) and subsequently activated by heating in the oven under normal pressure at 165 °C for 24 h. Zn@NDC_D and Zn_5TE@NDC_D were obtained as beige-coloured powders (23 mg and 18 mg, respectively). Yields: Zn@NDC_D 13%, Zn_5TE@NDC_D 9%.

**Synthesis of Zn_5TE_AuNPs@NDC/Zn_5TE_AuNCs@NDC**

Ultra-small AuNPs with a diameter of approximately 3 nm (Figure S8), coated with 1-octanethiol, were used to prepare composite materials. AuNPs were synthesised according to the procedure described in our earlier paper [45] based on the Brust-Schiffrin approach [71].

Gold nanoclusters (AuNCs) stabilised with L-cysteine and coated with polyethyleneimine (PEI) were prepared according to the protocol described by Abarghoei et al. [72], but modified, among others, by adding PEI as an additional stabilisation agent. Briefly, 92 mg of HAuCl_4_·3H_2_O was dissolved in 80 mL of ultra-pure water, and 84 mg of L-cysteine was added. The mixture was then stirred on a magnetic stirrer. The mixture, after the addition of cysteine, changed from yellow to white. After 5 min, 160 mg of PEI was added, and the flask containing the mixture was immersed in a thermostated oil bath set at 37 °C and stirred for 24 h. The oil bath temperature was maintained at 37 ± 2 °C. Immediately after the PEI addition, a white solid was precipitated and dissolved in a few minutes. The post-reaction mixture was opalescent and transparent, with no precipitated solids. The fabricated AuNCs were separated by water evaporation using a rotary vacuum evaporator and then dried in a vacuum oven (10 mbar, 40 °C) for 24 h. The product was a sticky yellow gel (290 mg, yield 97% calculated by Au content). The selected TEM images of the fabricated AuNPs and AuNCs are presented in the Supplementary Materials (Figure S8).

The synthesis of hybrid materials was similar to that of Zn-CPs (as described above), but 50 mg of AuNPs or 25 mg of AuNCs was also added. We employed both solvothermal and diffusion methods to fabricate hybrid materials. Thus, we applied the strategy of building the CPs framework in the presence of nanoparticles, thereby enabling the CPs to encapsulate the nanoparticles within the network. In the syntheses of Zn_5TE_AuNPs@NDC and Zn_5TE_AuNCs@NDC (from the solvothermal method), 125 mg and 134 mg were obtained with 49% and 58% yields, respectively.

**Catalytic assays**

Cycloaddition reactions with CO_2_ in the presence of the fabricated catalysts were performed in a pressure reactor constructed in our laboratory using commercially available stainless-steel components. The reactor was equipped with a manometer and valve system to introduce CO_2_ at the proper pressure (Figure S11). Reagents were placed in a Teflon vessel inside the reactor, and CO_2_ was introduced from a cylinder to achieve a starting pressure of 2 MPa. The reactor was immersed in a thermostated (±2 °C) oil bath on a magnetic stirrer.

The reactions were performed in the following system:

In a Teflon vessel, 7 mg of catalyst and 50 mg of TBAB (0.15 mmol) were placed; next, a suitable epoxide (14–25 mmol) was added. The vessel was closed in the reactor. Next, CO_2_ was introduced to the reactor at 2 MPa, and during the reaction, it was added to maintain a pressure of 1.5 MPa. The reactor was immersed in a thermostated oil bath maintained at the proper temperature and placed on a magnetic stirrer.

After a selected time, the reactor was cooled down using ice water and degassed. The post-reaction mixture was diluted with 15 mL of dichloromethane (DCM) or 15 mL of THF in the case of glycidol, for which weak solubility of the product in DCM was observed, and 300 µL of anisole (internal reference for GC analysis) was added; the obtained suspension was filtrated using a syringe filter (200 µm) or, in the case of catalyst recycling experiments, the catalyst was centrifuged from the post-reaction mixture (10 min, 10,000 rpm), allowing the catalyst to be reused. The recovered catalyst was washed with methanol, dried at 60 °C for 24 h, and reused. The mixture obtained after catalyst separation was analysed using gas chromatography (GC). Analyses of substrate conversion and product yields were performed using an internal standard procedure with anisole, added to the post-reaction mixture. For the selected post-reaction mixture (EPI, EPI-Br), products were isolated, and their identity was confirmed by ^1^H NMR spectroscopy (see the ESI). Products and unreacted reagents were isolated from the chosen post-reaction mixtures by passing them through a silica gel column using a hexane fraction from petroleum with ethyl acetate mixtures as the eluent.

For catalysts obtained by solvothermal and diffusion methods, the catalytic assay results were very similar, with higher yields from the solvothermal method. Therefore, we present results only for the catalytic materials obtained via the solvothermal procedure.

**Techniques**

X-ray photoelectron spectroscopy (XPS) analysis was performed using a PHI 5000 VersaProbe II Hybrid (Scanning ESCA Microprobe, ULVAC-PHI, Chigasaki, Japan), equipped with an Al Kα source (1486.6 eV, power 25 W). CasaXPS (version 2.3.19) software was used for the deconvolution of XPS signals. XPS data were calibrated using the binding energy of C 1s = 285.0 eV (C-C bond) as the internal standard [73].

**Scanning Electron Microscopy with Electron Dispersive Spectroscopy (SEM-EDS)**

The morphology and elemental composition of the fabricated nanocatalysts were analysed using the JEOL-JSM-5600 microscope (JEOL, Tokyo, Japan) with an OXFORD Link–ISIS–300 spectrometer (Zeiss, Jena, Germany). For imaging, the samples were coated with a thin Au–Pd alloy layer to improve conductivity.

**Transmission Electron Microscopy (TEM)**

Transmission electron microscopy (TEM) observations were carried out using a JEM-1400 JEOL Co. microscope at an acceleration voltage of 120 kV. The samples were obtained by casting an acetone suspension of the materials onto a carbon-coated copper microgrid (200 mesh) and then air-drying.

**Elemental Analyses**

The elemental UNIcube apparatus was used to analyse the ZnCPs samples and hybrid materials (C, H, N, S).

**An inductively coupled plasma mass spectrometry (ICP MS)**

An inductively coupled plasma mass spectrometer (ICP-MS), NexION 300D (PerkinElmer, Waltham, MA, USA), equipped with a quartz cyclonic spray chamber and Meinhard nebuliser, was used to determine the Zn and Au content.

**Surface areas and porosity (BET) analyses**

The adsorption–desorption isotherms of nitrogen (N_2_) and carbon dioxide (CO_2_) were measured, and specific surface area and pore volume analyses were performed using Micrometrics ASAP 2020. Before measuring, each sample was heated to 200 °C for 6 h; next, the pressure was reduced to less than 1 µmHg. The measurements of N_2_ adsorption were conducted at 77 K, while the adsorption of CO_2_ was at 273 K. The Brunauer–Emmett–Teller (BET) equation was used to calculate the surface area. Density functional theory (DFT) methods were used to calculate the pore volumes and their surfaces.

**NMR spectroscopy**

^1^H NMR spectra of the fabricated ZnCPs and hybrids were recorded after decomposition in deuterated sulphuric acid (100%) and after dissolving the products in DMSO-d6.

^1^H NMR spectra of post-reaction mixtures were recorded in CHCl_3_.

All spectra were recorded using the Bruker Corporation 500 MHz spectrometer (Bruker, Billerica, MA, USA).

The samples of MOFs with a mass of approximately 5 mg were first dissolved in 20 µL of D_2_SO_4_ (100%), and then 600 µL of DMSO-d6 was added. The suspension was sonicated for 15 min before recording a spectrum.

Zn_5TE@NDC

^1^H NMR (500 MHz, d6-DMSO) δ = 9.7 (s, COOH, 2H), 8.72–8.69 (m, CH, naphthalene ring 2H), 8.10 (s, CH, naphthalene ring, 2H), 8.05–7.93 (m, CH, naphthalene ring 2H), 7.67–7.64 (m, CH, naphthalene ring 2H), 7.09–7.52 (m, NH, tetrazole, naphthalene ring), 3.54 (s, H_2_O)

Zn_5TE_AuNPs@NDC/Zn_5TE_AuNCs@NDC

^1^H NMR (500 MHz, d6-DMSO) δ = 9.7 (s, COOH, 2H), 8.72–8.69 (m, CH, naphthalene ring 2H), 8.07 (s, CH, naphthalene ring, 2H), 8.07–7.91 (m, CH, naphthalene ring 2H), 7.67–7.62 (m, CH, naphthalene ring 2H), 7.02–6.8 (m, NH, tetrazole, naphthalene ring), 3.54 (s, H_2_O)

**Thermogravimetric analyses (TGA)**

TG analyses were conducted under an N_2_ atmosphere with a heating rate of 10 °C min^−1^ from 30 °C to 1000 °C using a TA Instruments Q50 V20.10 Build 36 thermogravimetric analyser (TA Instruments, Mokena, IL, USA).

**Gas chromatography (GC)**

GC analyses to determine yields and conversions of reactions with CO_2_ were performed using a Shimadzu GC2010 Plus gas chromatograph equipped with a split-mode capillary injection system and a flame ionisation detector (FID), capillary column: HP-5 (30 m × 0.320 mm, 0.25 μm, Agilent Technologies, Santa Clara, CA, USA).

Chromatography conditions: carrier gas—nitrogen (~80 kPa); split ratio: 50; injector temperature 250 °C; detector temperature 280 °C.

GC analysis method for the reaction of styrene oxide or propylene oxide with CO_2_:

Pressure: 80.9 kPa; Column flow: 2.43 mL/min; Oven temperature: Initial temp. 80 °C hold for 2 min; ramp rate 40 °C/min to 280 °C; final temp. 280 °C hold for 5 min (total of 12 min).

GC analysis method for the reaction of glycidol, epichlorohydrin and epibromohydrin with CO_2_:

Pressure: 77.3 kPa; Column flow: 2.54 mL/min; Oven temperature: Initial temp. 60 °C hold for 2 min; ramp rate 40 °C/min to 280 °C; final temp. 280 °C hold for 5 min (total 12.5 min).

**Powder X-ray diffraction (PXRD)**

Diffractograms were collected for CP samples deposited on <911>-oriented zero-background silicon wafers using a D8 Discover powder X-ray diffractometer (Bruker Inc., Billerica, MA, USA) equipped with a collimated Cu Kα radiation source (λ = 0.154 nm). Measurements were performed in the 2θ range of 3–60° in locked-coupled mode.

## 4. Conclusions

New coordination polymers were synthesised using two blocks, 2,4-NDC and 5-Atz, in a 1:1 molar ratio, yielding a high concentration of N-doped centres. The hybrid materials of the developed ZnCPs with gold nanoparticles and gold nanoclusters were fabricated using the “bottle around the ship” strategy, which involves solvothermal and diffusion methods. The last was performed at room temperature.

The designed nanomaterials were successfully applied as heterogeneous catalysts in the selective conversion of CO_2_ into cyclic organic carbonates (COCs). 5-Atz in the structures yields significantly better catalytic performance than the material constructed only from 1,4-NDC. Due to the high activity of nitrogen-rich moieties, this framework can play additional nucleophilic roles in the epoxide ring-opening process during CO_2_ fixation.

The developed nanocatalysts enable the synthesis of COCs under mild conditions (80 °C, 1.5 MPa CO_2_, solvent-free) with excellent yields (up to 96%) and selectivities (up to 100%), accompanied by high turnover frequencies (TOFs) of up to 408 h-1. Among the tested nanocatalysts, Zn_5TE_AuNCs@NDC, which was prepared by encapsulating gold nanoclusters, yielded the best results in catalytic assays with less active epoxides. The yield of propylene carbonate increased from 68% to 90%, and that of styrene carbonate rose from 69% to 94% when this hybrid catalyst was used, compared with the Zn_5TE@NDC catalyst. The designed catalysts can be recycled and reused without loss of activity.

The proposed approach contributes to the overall sustainability of the CO_2_ utilisation process using epoxides.

## Figures and Tables

**Table 1:** The elemental composition of the fabricated materials (given in % *w*/*w*) and the calculated empirical formulas.

Zn	C	N	S	H	O	Au
Zn_5TE@NDC						
10.5 ^a^ 11.9 ^d^	35.2 ^b^ 45.8 ^d^	18.9 ^b^ 15.2 ^d^	-	2.90 ^b^	32.5 ^c^ 26.3 ^d^	**-**
Formula	Zn^2+^ (NDC^2−^)_1.37_(TE)^+^_0.75_(TE)_0.92_• 7 H_2_O M = 632 u					
Zn_5TE_AuNPs@NDC						
14.2 ^a^ 29.6 ^d^	40.0 ^b^ 57.5 ^d^	17.4 ^b^ 16.5 ^d^	1.12 ^b^ 0.57 ^d^	3.69 ^b^	23.1 ^c^ 24.6 ^d^	0.52 ^a^ 0.23 ^d^
Formula	Zn^2+^ (NDC^2−^)_1.18_ (TE)^+^_0.35_(TE)_0.78_•0.01 Au•0.16 S•2 H_2_O M = 458 u					
Zn_5TE_AuNCs@NDC						
24.5 ^a^ 19.0 ^d^	35.4 ^b^ 39.6 ^d^	20.6 ^b^ 16.4 ^d^	1.85 ^b^ 1.94 ^d^	3.61 ^b^	11.54 ^c^ 16.65 ^d^	2.50 ^a^ 3.34 ^d^
Formula	Zn^2+^ (NDC^2−^)_0.58_ (OH)^−^_0.82_(TE)_0.78_• 0.03 Au • 0.15 S M = 284 u					

^a^ ICP MS; ^b^ EA; ^c^ calculated as 100% − (Zn% ^a^ + C% ^b^ + N% ^b^ + H% ^b^); ^d^ SEM-EDS, NDC^2−^ –deprotonated 1,4-naphtalene dicarboxylic acid, TE^+^ protonated 5-Atz, TE-5-Atz.

**Table 2:** XPS binding energies and elements’ concentrations from XPS for Zn_5TE_AuNCs@NDC.

Orbital	BDE [eV]	FWHM [eV]	Concentration [Weight%]	Concentration [Atom%]
C1s	285.0	1.320	19.8	29.4
	285.7	1.146	3.50	5.30
	286.4	1.536	10.9	16.1
	288.7	1.742	2.20	3.30
			36.4 ^a^	54.1 ^a^
N 1s	399.3	1.164	1.10	1.40
	400.5	1.326	8.50	10.8
	402.0	1.435	0.60	0.80
			10.2 ^a^	13.0 ^a^
Zn 2p_3/2_	1022.7	1.863		
Zn 3p_3/2_	89.60	2.735	22.7	6.2
Zn 3p_1/2_	92.6	3.453		
Au 4f_7/2_ Au 4f_5/2_	84.7 88.4	1.438 1.638	4.2	0.4
S 2p_3/2_	163.1 169.1	2.764 2.550	1.2 2.1	2.2 3.7
			3.3 ^a^	5.9 ^a^
O 1s	532.1 533.3	1.740 1.690	17.7 3.00	19.7 3.30
			20.7 ^a^	23.0 ^a^

^a^ global content.

**Table 3:** Porosity parameters determined from adsorption measurements at 273 K.

	S_0_ [m^2^ g^−1^] ^a^	V_0_ [cm^3^ g^−1^] ^a^	L_0_ [nm] ^b^	E_0_ [kJ mol^−1^] ^c^
Zn@NDC	146	0.079	1.09	21
Zn_5TE@NDC	67.7	0.038	1.13	21
Zn_5TE_AuNPs@NDC	34.3	0.026	1.55	18
Zn_5TE_AuNCs@NDC	23.3	0.021	1.82	17

^a^ from BET. ^b^ average pore widths according to Stoeckli et al. [64]. ^c^ energy of adsorption from the Dubinin equation [65].

**Table 4:** Results of catalytic assays for model epoxides using the fabricated catalysts ^a^.

Substrate	Product	Catalyst	Time [h]	Yield % ^b^	Conversion % ^b^	TON ^g^	TOF [h^−1^] ^h^
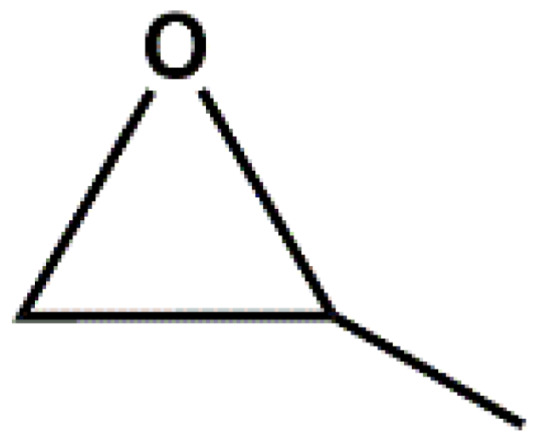	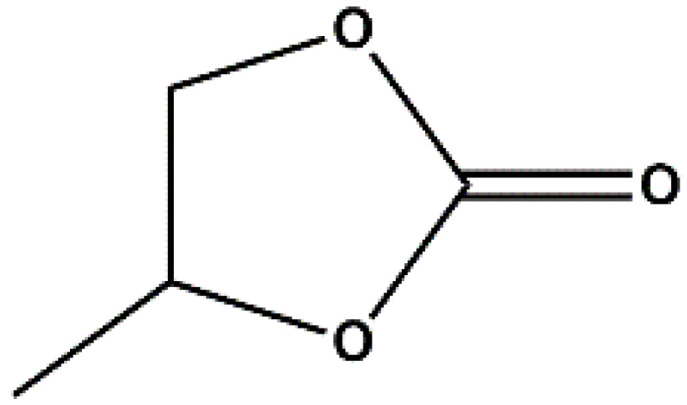	Zn@NDC Zn_5TE@NDC Zn_5TE_AuNPs@NDC ^e^ Zn_5TE_AuNCs@NDC ^f^	24	48 68 86 90	n.d. ^c^	1083 1145 1822 2160	45 48 76 90
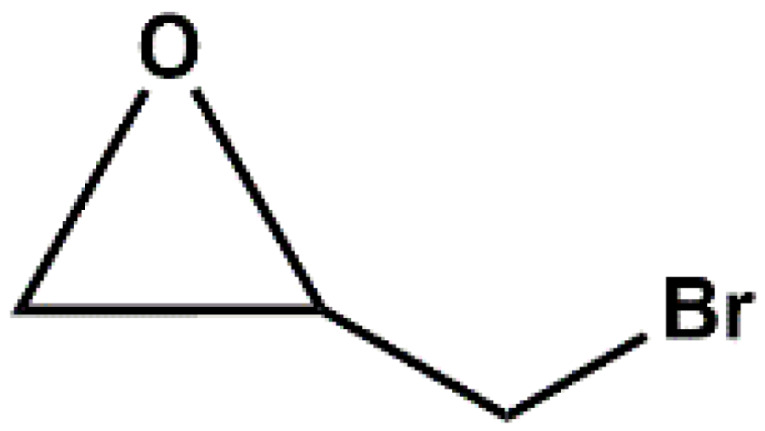	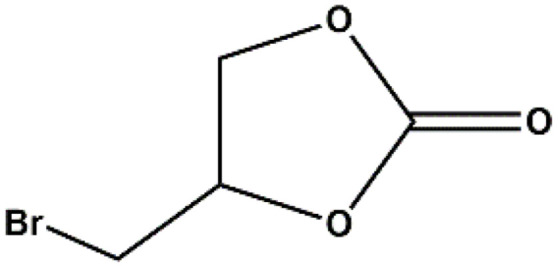	Zn_5TE@NDC Zn_5TE_AuNPs@NDC Zn_5TE_AuNCs@NDC	3	96 96, 87 ^d^ 96	97 97 97	1225 805 567	408 268 189
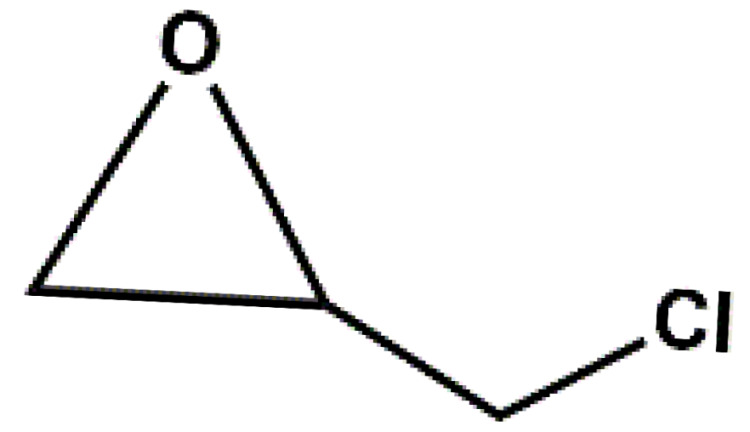	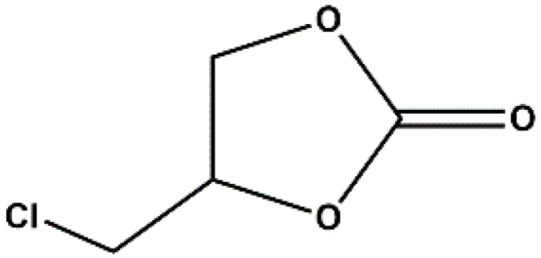	Zn_5TE@NDC Zn_5TE_AuNPs@NDC Zn_5TE_AuNCs@NDC	20	89 98 97	90 100 100	1110 1230 1216	55 61 61
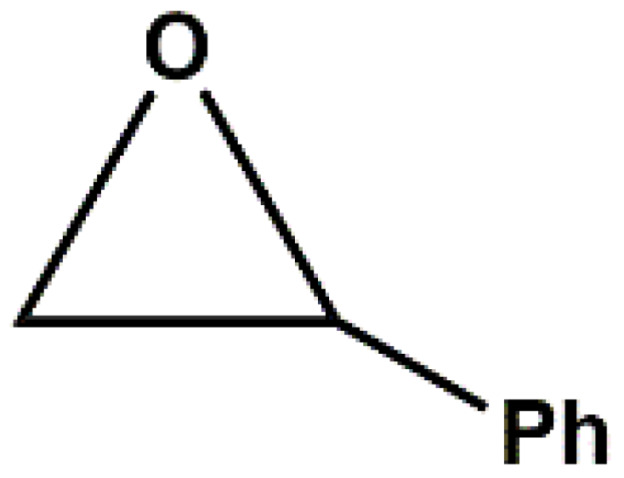	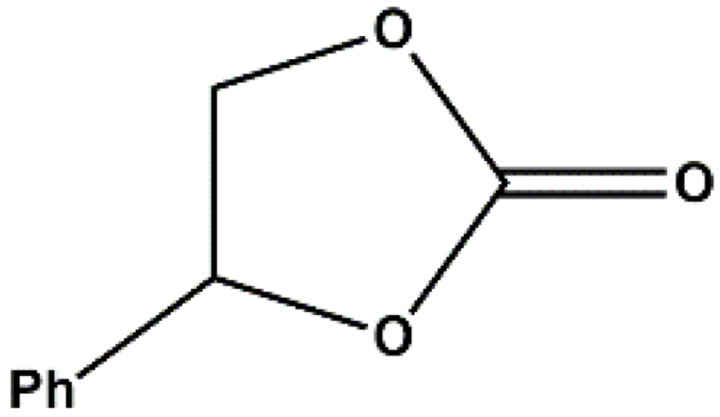	Zn_5TE@NDC Zn_5TE_AuNPs@NDC Zn_5TE_AuNCs@NDC	24	69 93 94	78 97 100	775 1020 1080	32 42 45
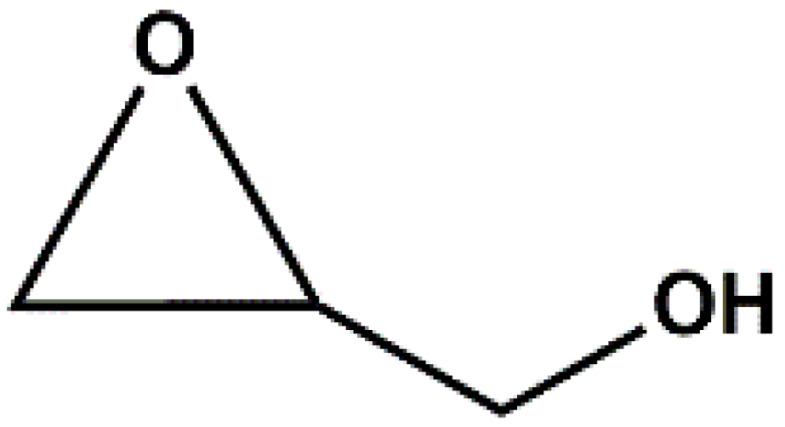	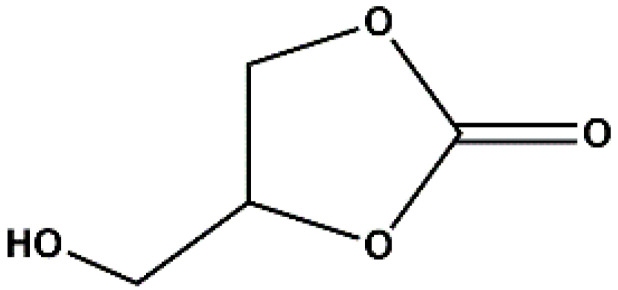	Zn_5TE@NDC Zn_5TE_AuNPs@NDC Zn_5TE_AuNCs@NDC	4	49 70 85	79 85 100	749 1200 892	187 195 223

^a^ Reaction conditions: 80 °C, 1.5 MPa CO_2_, without solvent, in a scale of 12 to 20 mmol of epoxide with 7 mg of the catalyst (0.1–0.2 mol%) and 50 mg (0.155 mmol, 1.2% mol) of TBAB; ^b^ determined by GC; ^c^ n.d. not determined because of the high volatility of propylene oxide; thus, it cannot be analysed using GC; ^d^ It was determined for isolated and purified product; ^e^ gold content 0.5% (ICP MS); ^f^ gold content 2.5% (ICP MS); ^g^ TON = (moles of product)/moles of catalyst); ^h^ TOF = TON/time.

## Data Availability

The authors have reviewed and edited the output and take full responsibility for the content of this publication.

## Supplementary Materials

The information can be downloaded at: https://www.mdpi.com/article/10.3390/molecules30244777/s1. Figure S1 to Figure S18, Tables S1 and S2 [74,75,76,77,78,79].

## References

[1] Ludwig J.R., Schindler C.S. (2017). Catalyst: Sustainable catalysis. Chem.

[2] Ciriminna R., Pagliaro M., Luque R. (2021). Heterogeneous catalysis under flow for the 21st century fine chemical industry. Green Energy Environ..

[3] Ciriminna R., Formenti M., Pagliaro M., Della Pina C. (2023). Practical aspects concerning catalysis with molecularly doped metals. ChemCatChem.

[4] Parangi T. (2025). Heterogeneous catalysis: An alternative approach for energy and environment. Rev. Inorg. Chem..

[5] Engel E.R., Scott J.L. (2020). Advances in the green chemistry of coordination polymer materials. Green Chem..

[6] Lin Z., Richardson J.J., Zhou J., Caruso F. (2023). Direct synthesis of amorphous coordination polymers and metal–organic frameworks. Nat. Rev. Chem..

[7] Biradha K., Das S.K., Bu X.-H. (2022). Coordination polymers as heterogeneous catalysts for water splitting and CO_2_ fixation. Cryst. Growth Des..

[8] Gupta A.K., Guha N., Krishnan S., Mathur P., Rai D.K. (2020). A Three-Dimensional Cu(II)-MOF with Lewis acid−base dual functional sites for Chemical Fixation of CO_2_ via Cyclic Carbonate Synthesis. J. CO2 Util..

[9] Wang Y., Astruc D., Abd-El-Aziz A.S. (2019). Metallopolymers for advanced sustainable applications. Chem. Soc. Rev..

[10] Roy D., Kumar P., Soni A., Nemiwal M. (2023). A versatile and microporous Zn-based MOFs as a recyclable and sustainable heterogeneous catalyst for various organic transformations: A review (2015-present). Tetrahedron.

[11] Vasile Scaeteanu G., Maxim C., Badea M., Olar R. (2023). Zinc (II) Carboxylate Coordination Polymers with Versatile Applications. Molecules.

[12] San Sebastian E., Rodríguez-Diéguez A., Seco J.M., Cepeda J. (2018). Coordination Polymers with Intriguing Photoluminescence Behavior: The Promising Avenue for Greatest Long-Lasting Phosphors. Eur. J. Inorg. Chem..

[13] Ali S.M., Appolloni A., Cavallaro F., D’Adamo I., Di Vaio A., Ferella F., Gastaldi M., Ikram M., Kumar N.M., Martin M.A. (2023). Development goals towards sustainability. Sustainability.

[14] Alper E., Orhan O.Y. (2017). CO_2_ utilization: Developments in conversion processes. Petroleum.

[15] Grignard B., Gennen S., Jérôme C., Kleij A.W., Detrembleur C. (2019). Advances in the use of CO_2_ as a renewable feedstock for the synthesis of polymers. Chem. Soc. Rev..

[16] Álvarez A., Bansode A., Urakawa A., Bavykina A.V., Wezendonk T.A., Makkee M., Gascon J., Kapteijn F. (2017). Challenges in the greener production of formates/formic acid, methanol, and DME by heterogeneously catalyzed CO_2_ hydrogenation processes. Chem. Rev..

[17] Song J., Liu Q., Liu H., Jiang X. (2018). Recent Advances in Palladium-Catalyzed Carboxylation with CO_2_. Eur. J. Org. Chem..

[18] Zhang Z., Ju T., Ye J.-H., Yu D.-G. (2017). CO_2_ = CO+ O: Redox-neutral lactamization and lactonization of C–H bonds with CO_2_. Synlett.

[19] Hu J., Yu L., Deng J., Wang Y., Cheng K., Ma C., Zhang Q., Wen W., Yu S., Pan Y. (2021). Sulfur vacancy-rich MoS_2_ as a catalyst for the hydrogenation of CO_2_ to methanol. Nat. Catal..

[20] Yan T., Liu H., Zeng Z., Pan W. (2023). Recent progress of catalysts for synthesis of cyclic carbonates from CO_2_ and epoxides. J. CO2 Util..

[21] Kamphuis A.J., Picchioni F., Pescarmona P.P. (2019). CO_2_-fixation into cyclic and polymeric carbonates: Principles and applications. Green Chem..

[22] Pescarmona P.P. (2021). Cyclic carbonates synthesised from CO_2_: Applications, challenges and recent research trends. Curr. Opin. Green Sustain. Chem..

[23] Song X., Wang J., Yang L., Pan H., Zheng B. (2020). The transformation strategies between homogeneous and heterogeneous catalysts for the coupling reactions of CO_2_ and epoxides/olefins. Inorg. Chem. Commun..

[24] Sopeña S., Laserna V., Guo W., Martin E., Escudero-Adán E.C., Kleij A.W. (2016). Regioselective organocatalytic formation of carbamates from substituted cyclic carbonates. Adv. Synth. Catal..

[25] Zubar V., Lebedev Y., Azofra L.M., Cavallo L., El-Sepelgy O., Rueping M. (2018). Hydrogenation of CO_2_-Derived Carbonates and Polycarbonates to Methanol and Diols by Metal–Ligand Cooperative Manganese Catalysis. Angew. Chem. Int. Ed..

[26] Nguyen P.T., Tran Y.B. (2021). Copper-based metal-organic framework for selective CO_2_ adsoprtion and catalysis fixation of CO_2_ into cyclic carbonates. ChemistrySelect.

[27] Motokucho S., Takenouchi Y., Satoh R., Morikawa H., Nakatani H. (2020). Novel polyurethane-catalyzed cyclic carbonate synthesis using CO_2_ and epoxide. ACS Sustain. Chem. Eng..

[28] Ye S., Wang W., Liang J., Wang S., Xiao M., Meng Y. (2020). Metal-free approach for a one-pot construction of biodegradable block copolymers from epoxides, phthalic anhydride, and CO_2_. ACS Sustain. Chem. Eng..

[29] Pander M., Janeta M., Bury W. (2021). Quest for an Efficient 2-in-1 MOF-Based Catalytic System for Cycloaddition of CO_2_ to Epoxides under Mild Conditions. ACS Appl. Mater. Interfaces.

[30] Beyzavi M.H., Stephenson C.J., Liu Y., Karagiaridi O., Hupp J.T., Farha O.K. (2015). Metal–organic framework-based catalysts: Chemical fixation of CO_2_ with epoxides leading to cyclic organic carbonates. Front. Energy Res..

[31] Pal T.K., De D., Bharadwaj P.K. (2020). Metal–organic frameworks for the chemical fixation of CO_2_ into cyclic carbonates. Coord. Chem. Rev..

[32] Al-Rowaili F.N., Zahid U., Onaizi S., Khaled M., Jamal A., AL-Mutairi E.M. (2021). A review for Metal-Organic Frameworks (MOFs) utilization in capture and conversion of carbon dioxide into valuable products. J. CO2 Util..

[33] Tran Y.B.N., Nguyen P.T.K., Luong Q.T., Nguyen K.D. (2020). Series of M-MOF-184 (M = Mg, Co, Ni, Zn, Cu, Fe) Metal–Organic Frameworks for Catalysis Cycloaddition of CO_2_. Inorg. Chem..

[34] Agarwal R.A., Gupta A.K., De D. (2019). Flexible Zn-MOF Exhibiting Selective CO_2_ Adsorption and Efficient Lewis Acidic Catalytic Activity. Cryst. Growth Des..

[35] Eskemech A., Chand H., Karmakar A., Krishnan V., Koner R.R. (2024). Zn-MOF as a Single Catalyst with Dual Lewis Acidic and Basic Reaction Sites for CO_2_ Fixation. Inorg. Chem..

[36] Lan J., Qu Y., Wang Z., Xu P., Sun J. (2021). A facile fabrication of a multi-functional and hierarchical Zn-based MOF as an efficient catalyst for CO_2_ fixation at room-temperature. Inorg. Chem. Front..

[37] Kang X., Mei Z., Wang H., Gu J., Xue J., Azam M. (2024). Structural Diversity and Catalytic Properties of Coordination Polymers with Functionalized Naphthalene Dicarboxylate Linkers. Cryst. Growth Des..

[38] Yan Q., Lin Y., Wu P., Zhao L., Cao L., Peng L., Kong C., Chen L. (2013). Designed Synthesis of Functionalized Two-Dimensional Metal–Organic Frameworks with Preferential CO_2_ Capture. ChemPlusChem.

[39] Vaidhyanathan R., Iremonger S.S., Dawson K.W., Shimizu G.K.H. (2009). An amine-functionalized metal organic framework for preferential CO_2_ adsorption at low pressures. Chem. Commun..

[40] Chand H., Allasia N., Cipriano L.A., Liberto G.D., Kwon I.S., Zhang M., Pacchioni G., Krishnan V., Vilé G. (2025). Synergistic Effects in a Nitrogen-Coordinated Zinc Single-Atom Catalyst for Efficient CO_2_ Cycloaddition. ChemCatChem.

[41] Wu Y.-L., Tang P.-F., Zhang Q., Yan Y.-T., Zhang S., Yang G.-P., Wang Y.-Y. (2024). Nanoarchitectonics and catalytic performances of metal–organic frameworks supported metal nanoparticles. Appl. Organomet. Chem..

[42] Wang Y., Ling L., Zhang W., Guo J., Ding K., Duan W., Liu B. (2019). “Ship-in-Bottle” Strategy to Encapsulate Shape-Controllable Metal Nanocrystals into Metal–Organic Frameworks: Internal Space Matters. Chem. Mater..

[43] Sanchis-Gual R., Coronado-Puchau M., Mallah T., Coronado E. (2023). Hybrid nanostructures based on gold nanoparticles and functional coordination polymers: Chemistry, physics and applications in biomedicine, catalysis and magnetism. Coord. Chem. Rev..

[44] Sankar M., He Q., Engel R.V., Sainna M.A., Logsdail A.J., Roldan A., Willock D.J., Agarwal N., Kiely C.J., Hutchings G.J. (2020). Role of the support in gold-containing nanoparticles as heterogeneous catalysts. Chem. Rev..

[45] Kopacka G., Wasiluk K., Majewski P.W., Kopyt M., Kwiatkowski P., Megiel E. (2024). Aluminium-Based Metal–Organic Framework Nano Cuboids and Nanoflakes with Embedded Gold Nanoparticles for Carbon Dioxide Fixation with Epoxides into Cyclic Esters. Int. J. Mol. Sci..

[46] Megiel E. (2017). Surface modification using TEMPO and its derivatives. Adv. Colloid Interface Sci..

[47] Swiech O., Bilewicz R., Megiel E. (2013). TEMPO coated Au nanoparticles: Synthesis and tethering to gold surfaces. RSC Adv..

[48] Witzel S., Hashmi A.S.K., Xie J. (2021). Light in gold catalysis. Chem. Rev..

[49] Zhang W., Bojdys M.J., Pinna N. (2023). A universal synthesis strategy for tunable metal-organic framework nanohybrids. Angew. Chem. Int. Ed..

[50] Bozack M., Zhou Y., Worley S. (1994). Structural modifications in the amino acid lysine induced by soft x-ray irradiation. J. Chem. Phys..

[51] Bikas R., Heydari N., Lis T. (2023). Catalytic synthesis of tetrazoles by a silica supported Zn (II) coordination compound containing azide ligand. J. Mol. Struct..

[52] Hu P., Iyoki K., Yamada H., Yanaba Y., Ohara K., Katada N., Wakihara T. (2019). Synthesis and characterization of MFI-type zincosilicate zeolites with high zinc content using mechanochemically treated Si–Zn oxide composite. Microporous Mesoporous Mater..

[53] Jedras A., Matusik J., Dhanaraman E., Fu Y.-P., Cempura G. (2024). Tuning the structural and electronic properties of Zn–Cr LDH/GCN heterostructure for enhanced photodegradation of estrone in UV and visible light. Langmuir.

[54] Cheng Y., Lu S., Xu W., Wen H., Wang J. (2015). Fabrication of superhydrophobic Au–Zn alloy surface on a zinc substrate for roll-down, self-cleaning and anti-corrosion properties. J. Mater. Chem. A.

[55] Wertheim G.K., Campagna M., Hüfner S. (1974). Density of states of Zn and β-Brass. Phys. Condens. Matter.

[56] Thomas T.D., Weightman P. (1986). Valence electronic structure of AuZn and AuMg alloys derived from a new way of analyzing Auger-parameter shifts. Phys. Rev. B.

[57] Hostetler M.J., Wingate J.E., Zhong C.-J., Harris J.E., Vachet R.W., Clark M.R., Londono J.D., Green S.J., Stokes J.J., Wignall G.D. (1998). Alkanethiolate gold cluster molecules with core diameters from 1.5 to 5.2 nm: Core and monolayer properties as a function of core size. Langmuir.

[58] Kaim A., Szydłowska J., Piotrowski P., Megiel E. (2012). One-pot synthesis of gold nanoparticles densely coated with nitroxide spins. Polyhedron.

[59] Riga J., Snauwaert P., De Pryck A., Lazzaroni R., Boutique J.P., Verbist J.J., Brédas J.L., André J.M., Taliani C. (1987). Electronic structure of sulphur-containing conducting polymers. Synth. Met..

[60] White T.W., Duncan D.A., Fortuna S., Wang Y.L., Moreton B., Lee T.L., Blowey P., Costantini G., Woodruff D.P. (2018). A structural investigation of the interaction of oxalic acid with Cu(110). Surf. Sci..

[61] Taghavikish M., Subianto S., Dutta N.K., Roy Choudhury N. (2016). Novel Thiol-Ene Hybrid Coating for Metal Protection. Coatings.

[62] Chen K.-Y., Yan M., Luo K.-H., Wei Y., Yeh J.-M. (2022). Comparative Studies of the Dielectric Properties of Polyester Imide Composite Membranes Containing Hydrophilic and Hydrophobic Mesoporous Silica Particles. Materials.

[63] Zhao D., Liu X.-H., Guo J.-H., Xu H.-J., Zhao Y., Lu Y., Sun W.-Y. (2018). Porous Metal–Organic Frameworks with Chelating Multiamine Sites for Selective Adsorption and Chemical Conversion of Carbon Dioxide. Inorg. Chem..

[64] Stoeckli F., López-Ramón M., Hugi-Cleary D., Guillot A. (2001). Micropore sizes in activated carbons determined from the Dubinin-Radushkevich equation. Carbon.

[65] Chen S., Yang R. (1994). Theoretical basis for the potential theory adsorption isotherms. The Dubinin-Radushkevich and Dubinin-Astakhov equations. Langmuir.

[66] Maity S., Bain D., Patra A. (2019). An overview on the current understanding of the photophysical properties of metal nanoclusters and their potential applications. Nanoscale.

[67] Jin R., Li G., Sharma S., Li Y., Du X. (2021). Toward Active-Site Tailoring in Heterogeneous Catalysis by Atomically Precise Metal Nanoclusters with Crystallographic Structures. Chem. Rev..

[68] Pichugina D.y.A., Kuz’menko N.E., Shestakov A.F. (2015). Ligand-protected gold clusters: The structure, synthesis and applications. Russ. Chem. Rev..

[69] Meng B., Liu Y., Xing Y., Wang X., Li W. (2016). Octanuclear zinc (II) carboxylate framework based on 1, 4-naphthalenedicarboxylic acid. Inorg. Chem. Commun..

[70] Tang L., Zhang S., Wu Q., Wang X., Wu H., Jiang Z. (2018). Heterobimetallic metal–organic framework nanocages as highly efficient catalysts for CO_2_ conversion under mild conditions. J. Mater. Chem. A.

[71] Brust M., Walker M., Bethell D., Schiffrin D.J., Whyman R. (1994). Synthesis of thiol-derivatised gold nanoparticles in a two-phase liquid–liquid system. J. Chem. Soc. Chem. Commun..

[72] Abarghoei S., Fakhri N., Borghei Y.S., Hosseini M., Ganjali M.R. (2019). A colorimetric paper sensor for citrate as biomarker for early stage detection of prostate cancer based on peroxidase-like activity of cysteine-capped gold nanoclusters. Spectrochim. Acta Part A Mol. Biomol. Spectrosc..

[73] Finšgar M., Fassbender S., Hirth S., Milošev I. (2009). Electrochemical and XPS study of polyethyleneimines of different molecular sizes as corrosion inhibitors for AISI 430 stainless steel in near-neutral chloride media. Mater. Chem. Phys..

[74] Phan H., de la Cruz-Sánchez P., Cabrera-Afonso M.J., Martín-Matute B. (2025). Auto-relay catalysis for the oxidative carboxylation of alkenes into cyclic carbonates by a MOF catalyst. Green Chem..

[75] Liang J., Chen R.-P., Wang X.-Y., Liu T.-T., Wang X.-S., Huang Y.-B., Cao R. (2017). Postsynthetic ionization of an imidazole-containing metal-organic framework for the cycloaddition of carbon dioxide and epoxides. Chem. Sci..

[76] Song J., Zhang Z., Hu S., Wu T., Jiang T., Han B. (2009). MOF-5/n-Bu_4_NBr: An efficient catalyst system for the synthesis of cyclic carbonates from epoxides and CO_2_ under mild conditions. Green Chem..

[77] Cho H.-Y., Yang D.-A., Kim J., Jeong S.-Y., Ahn W.-S. (2012). CO_2_ adsorption and catalytic application of Co-MOF-74 synthesized by microwave heating. Catal. Today.

[78] Noh J., Kim Y., Park H., Lee J., Yoon M., Park M.H., Kim Y., Kim M. (2018). Functional Group Effects on a Metal-Organic Framework Catalyst for CO_2_ Cycloaddition. J. Ind. Eng. Chem..

[79] Senthilkumar S., Maru M.S., Somani R.S., Bajaj H.C., Neogi S. (2018). Unprecedented NH2-MIL-101(Al)/n-Bu4NBr system as solvent-free heterogeneous catalyst for efficient synthesis of cyclic carbonates via CO_2_ cycloaddition. Dalton Trans..

