# Supplementary material for: Eco-Friendly Coordination Polymers with Incorporated Nitrogen-Rich Heterocyclic Ligand and Their Hybrids with Gold Nanostructures for Catalytic Conversion of Carbon Dioxide
Source: Molecules. 2025 Dec 15;30(24):4777. doi: 10.3390/molecules30244777 (PMC12736215; doi:10.3390/molecules30244777)
Supplement: Supplementary file 1 [file molecules-30-04777-s001.zip › molecules-4023915-supplementary.pdf]

Eco-friendly coordination polymers with incorporated nitrogen-rich heterocyclic ligand and their hybrids with gold nanostructures for catalytic conversion of carbon dioxide

Kinga Wasiluk[1], Gabriela Kopacka,[1]Michał Kopyt,[1,2] Piotr Kwiatkowski,[1,2]Pawel W. Majewski [1,2] and Elżbieta Megiel[1*]

1 University of Warsaw, Faculty of Chemistry, Pasteur 1, 02-093 Warsaw, Poland E-mail: e.megiel2@uw.edu.pl
2 University of Warsaw, Biological and Chemical Research Centre, Żwirki i Wigury 101, 02-089 Warsaw, Poland

Supplementary Materials

Table of Contents

Figure S1. The XPS survey spectrum for Zn_5TE_AuNCs@NDC
Figure S2. HR-XPS S 2p and O 1s spectra for Zn_5TE_AuNCs@NDC
Figure S3. SEM images of the fabricated Zn-CPs and their composites with AuNPs and AuNCs
Figure S4. TEM images of the fabricated Zn-CPs and their composites with AuNPs and AuNCs
Figure S5. N_2 adsorption-desorption isotherms at 77 K for selected fabricated nanocatalysts
Figure S6. DFT pores' distribution determined from the CO_2 adsorption isotherms at 273 K

Figure S7. UV-Vis spectra recorded for gold nanostructures used for the fabrication of hybrid nanocatalysts
Figure S8. The selected TEM images of gold nanostructures used for the fabrication of composite nanocatalysts, and a statistical representation, were prepared based on these images
Figure S9. The yield of carbonate of epibromohydrin that was obtained in five subsequent cycles
Figure S10. Conversion vs. time for CO_2 fixation with epibromohydrin catalysed by the fabricated catalysts and first-order kinetic simulation curves
Figure S11. The picture of the reaction set used in the catalytic assays
Figure S12. Elemental maps of Zn, C, and N concentrations obtained from SEM-EDS analyses
The results of elemental analyses for the fabricated nanocatalysts
Fig.S13-S17. ^{1}H NMR analyses of post-reaction mixtures (after TBAB separation)
Table S1. Comparison of Zn_5TE_AuNCs@NDC with similar reported catalysts
Figure S18. PXRD patterns of the CP building blocks and the obtained frameworks
Table S2 SEM-EDS analysis results for Zn_5TE_AuNCs@NDC after its use in the 5th catalytic assay.
References

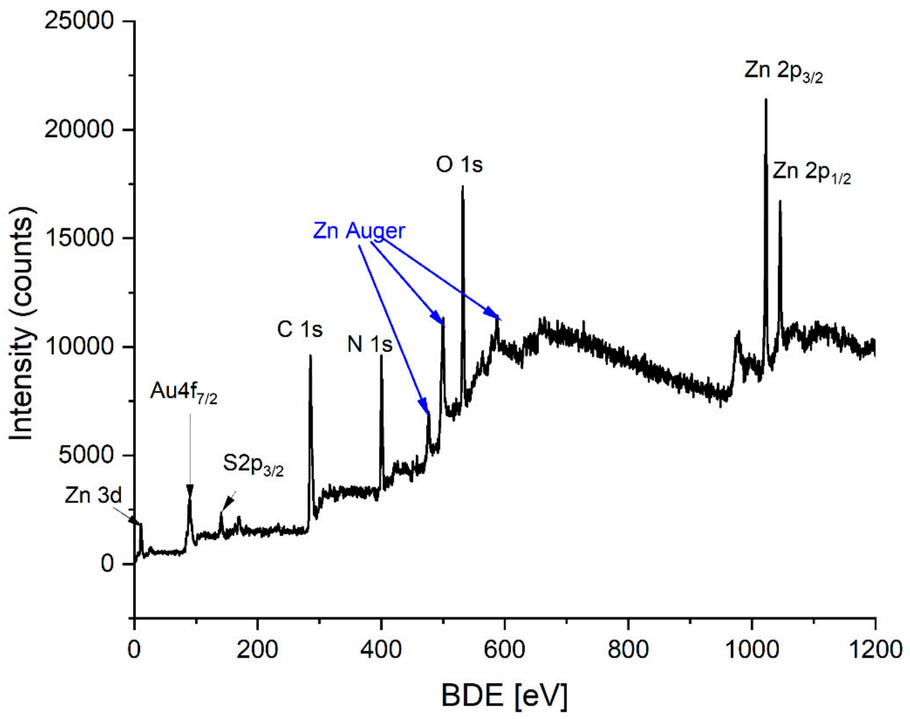

Figure S1. The XPS survey spectrum for Zn_5TE_AuNCs@NDC.

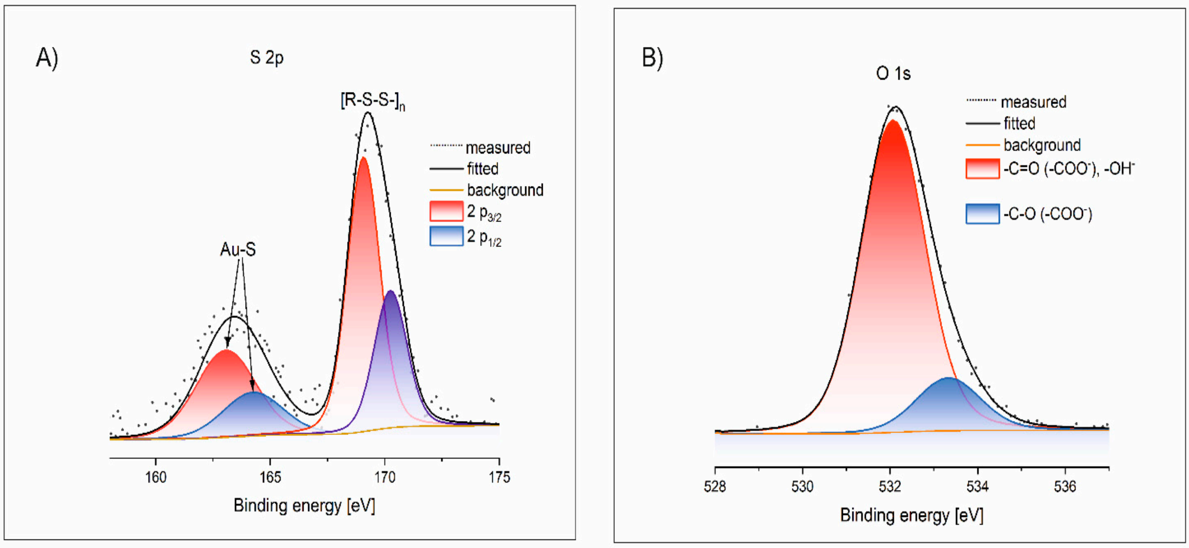

Figure S2. HR-XPS spectra S 2p and O 1s for Zn_5TE_AuNCs@NDC.

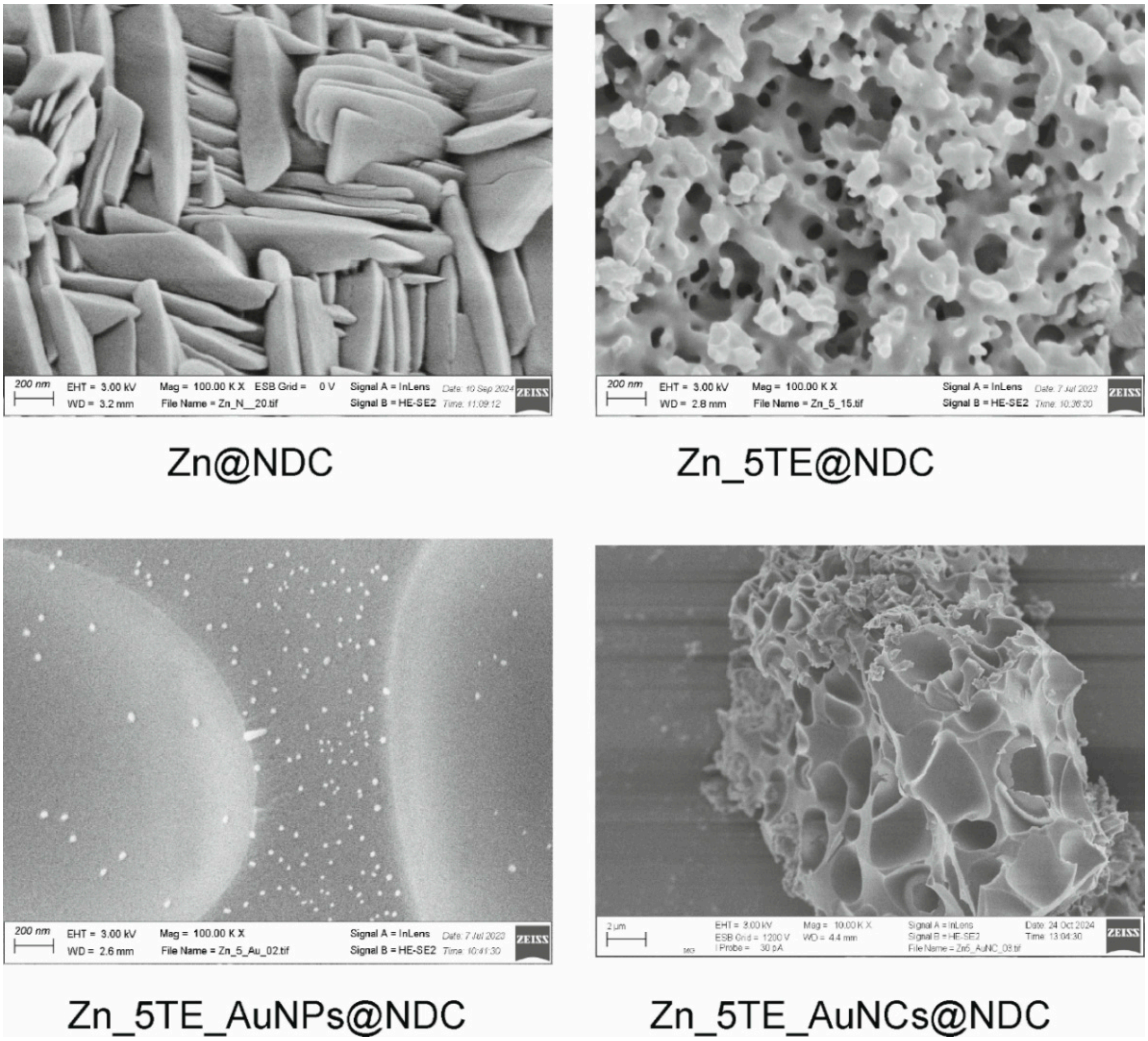

Figure S3. SEM images of the fabricated Zn-CPs and their composites with AuNPs and AuNCs.

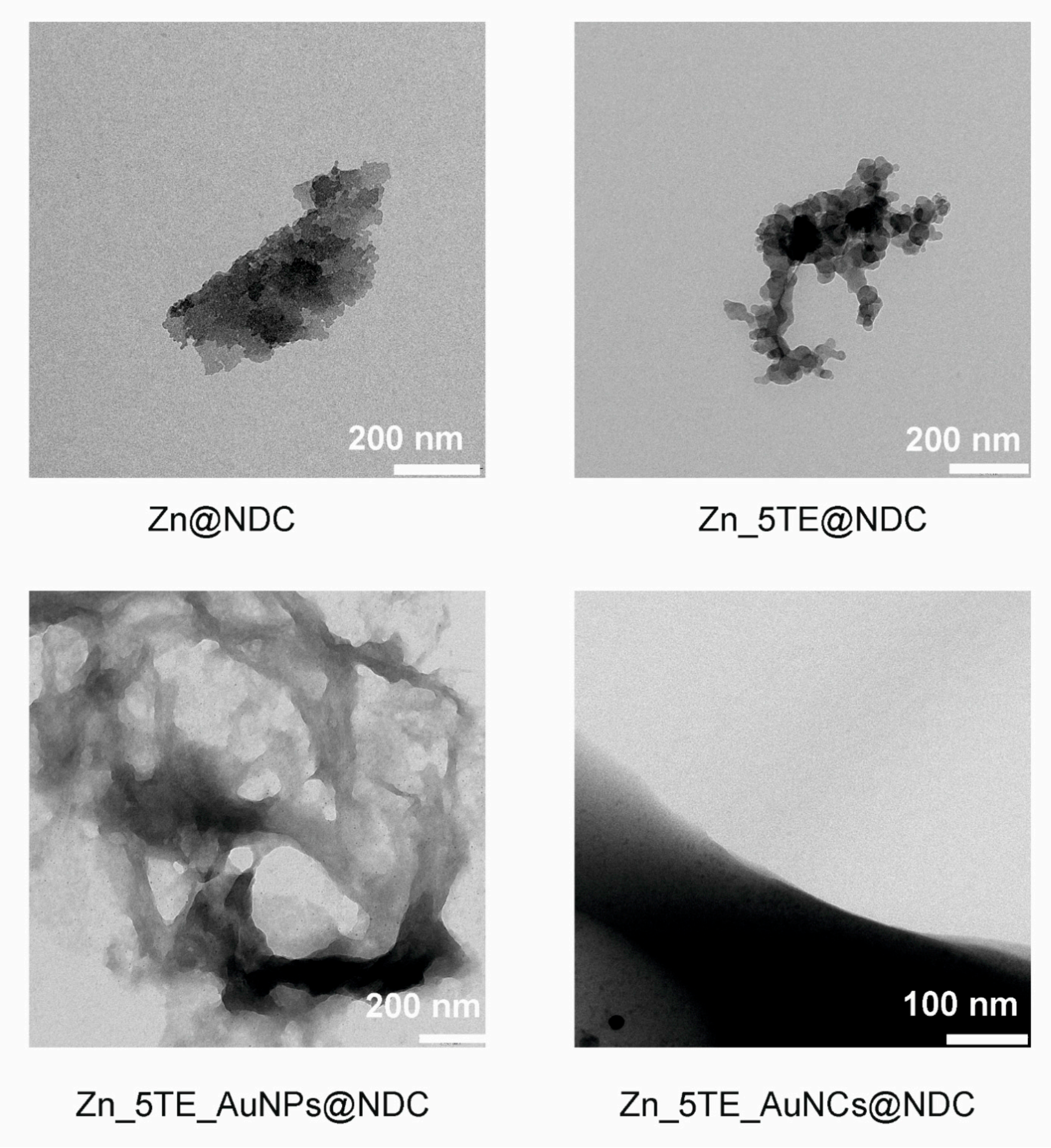

Figure S4. TEM images of the fabricated Zn-CPs and their composites with AuNPs and AuNCs.

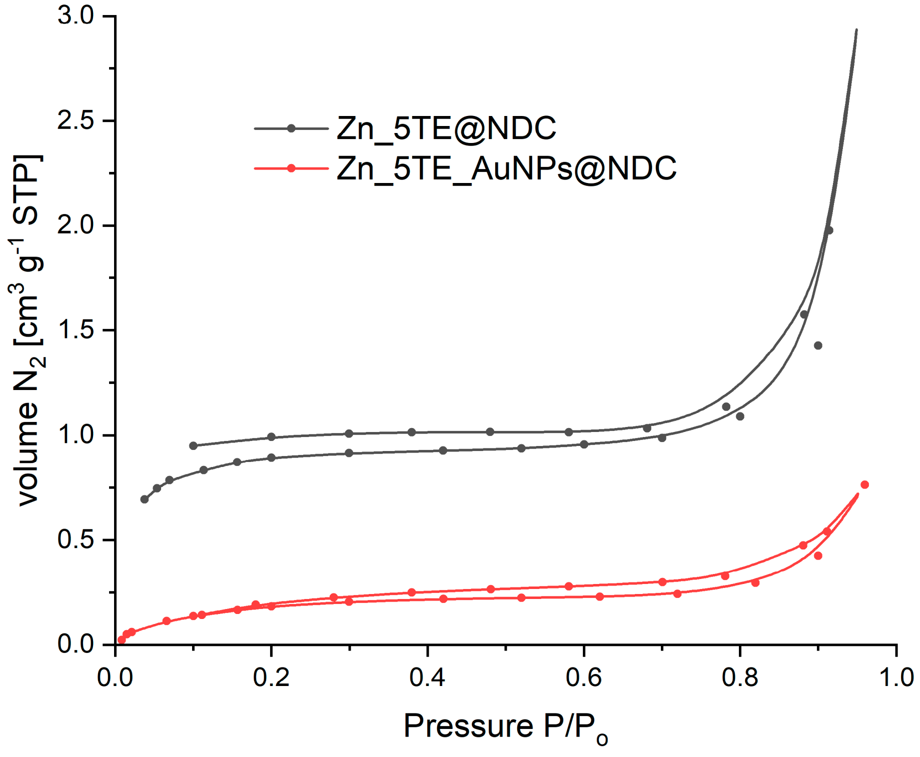

Figure S5. N_2 adsorption-desorption isotherms at 77 K for selected fabricated nanocatalysts.

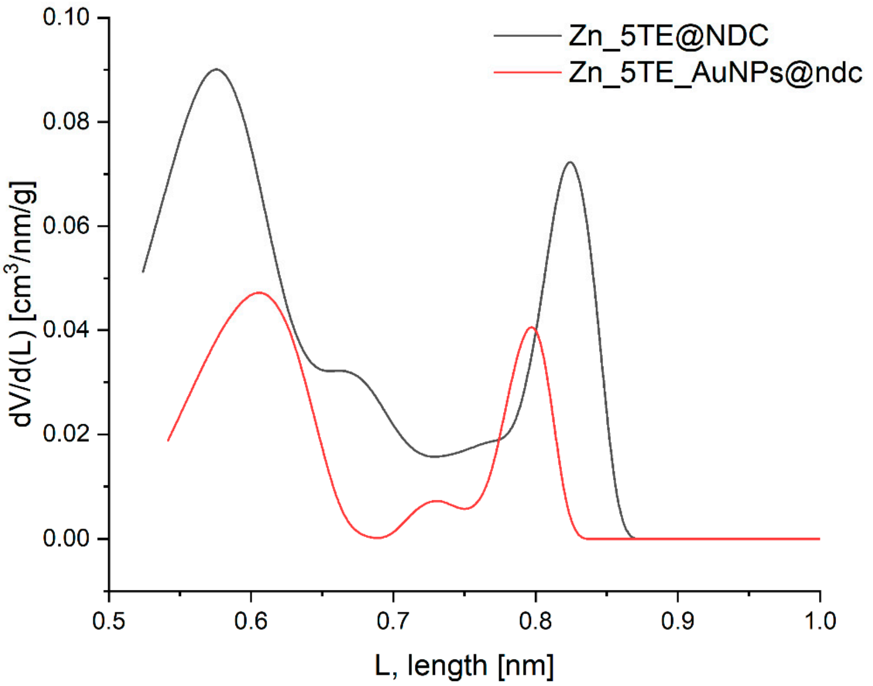

Figure S6. DFT pores' distribution determined from the CO_2 adsorption isotherms at 273 K.

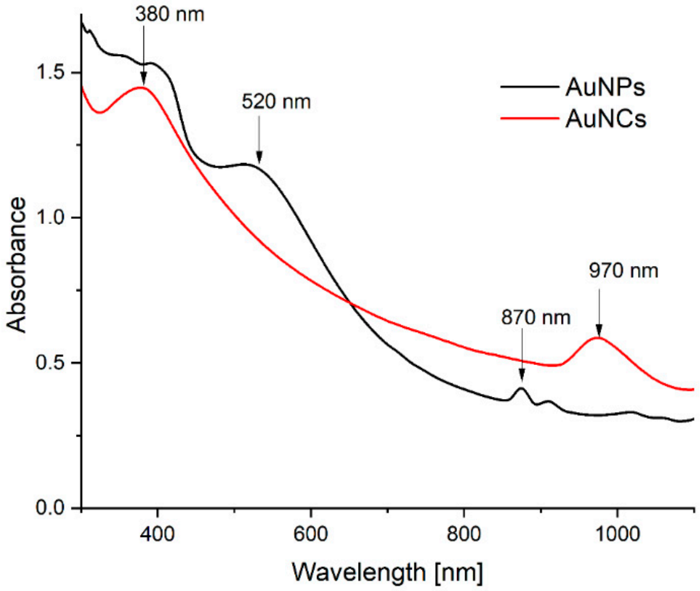

Figure S7. UV-Vis spectra recorded for gold nanostructures used for the fabrication of hybrid nanocatalysts.

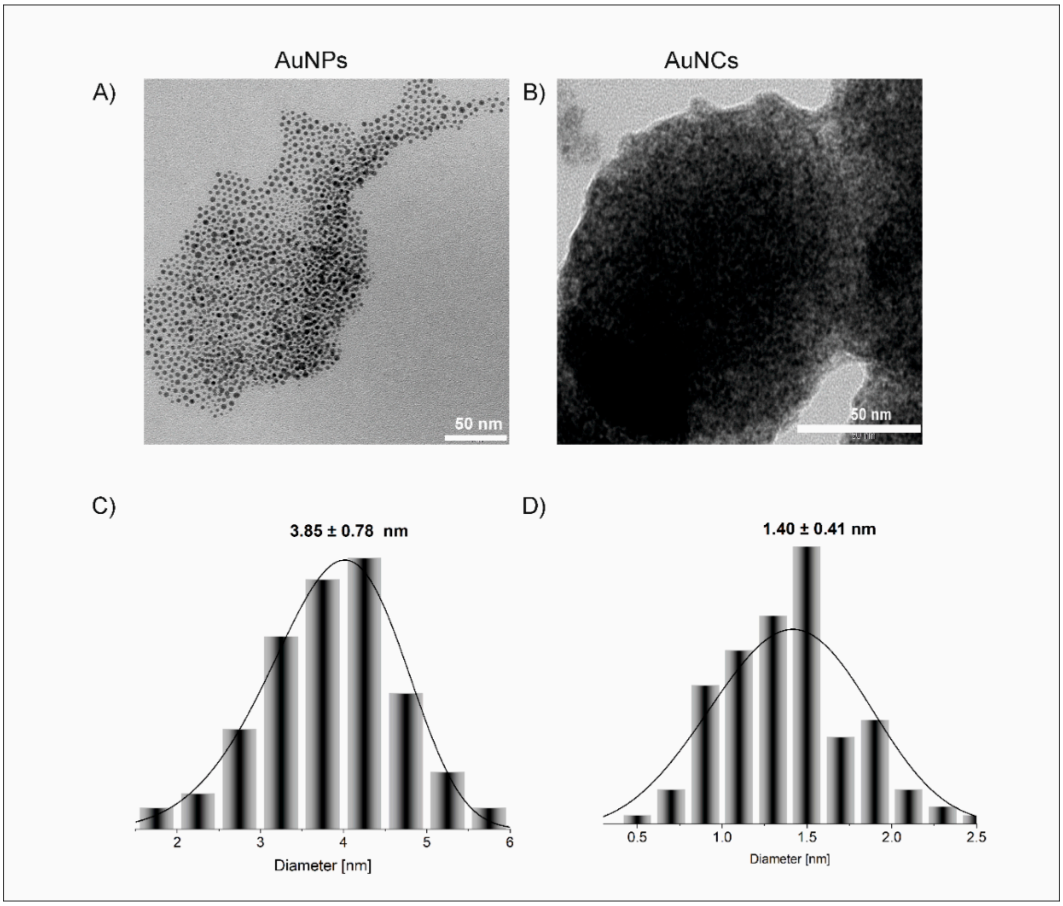

Figure S8. TEM images of gold nanostructures used to fabricate composite nanocatalysts: A) gold nanoparticles, B) gold nanoclusters and histograms, C) and D) prepared based on these images, respectively.

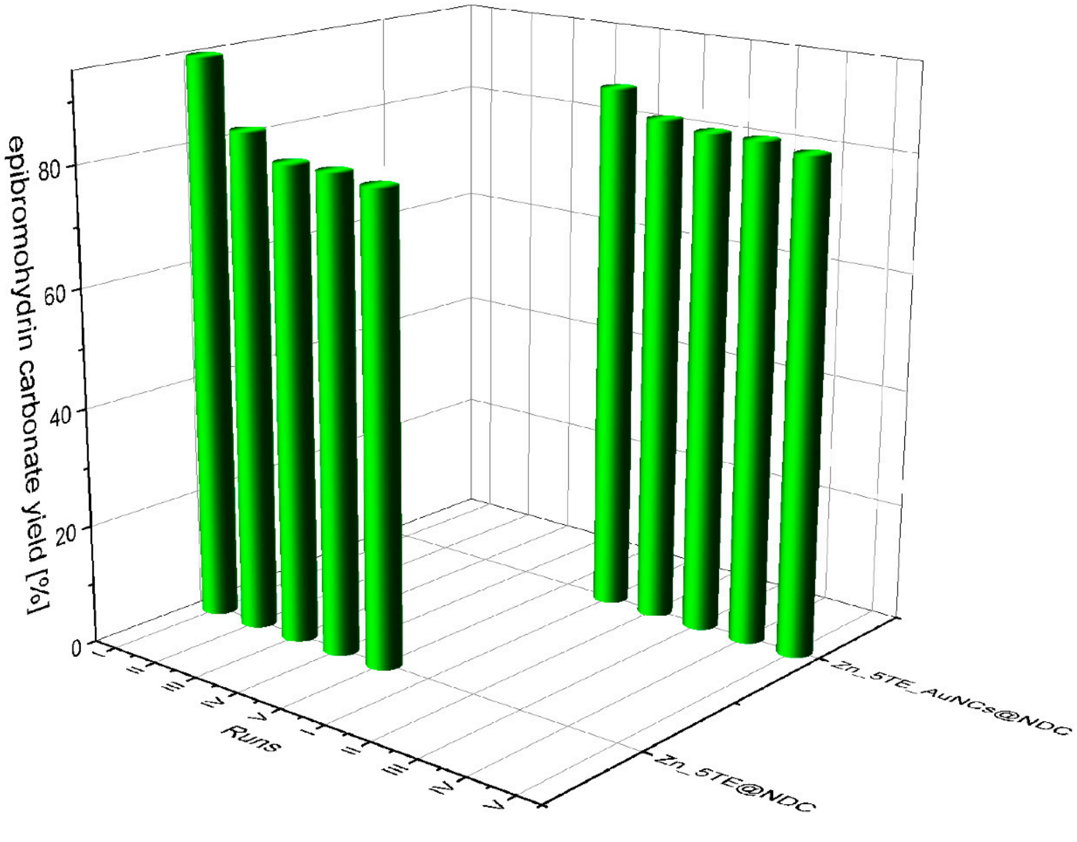

Figure S9. The yield of carbonate of epibromohydrin that was obtained in five subsequent cycles using two types of catalyst (2 h, 80°C, 1.5 MPa CO_2, 15 mg of catalysts in the first cycle, 50 mg of TBAB, 14 mmol of EPI-Br).

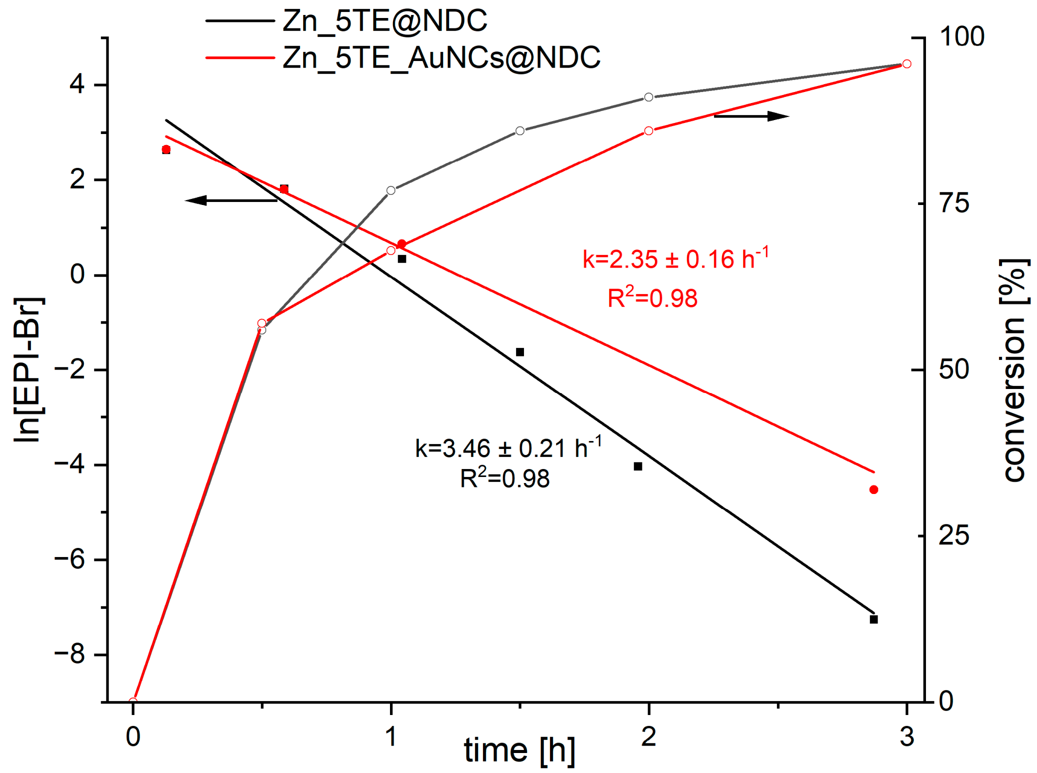

Figure S10. Conversion vs. time for CO_2 fixation with epibromohydrin catalysed by the fabricated catalysts and first-order kinetic simulation curves drawn in the same colour. Reaction conditions: 80 °C, 1.5 MPa CO_2, 7 mg of catalyst, 50 mg TBAB.

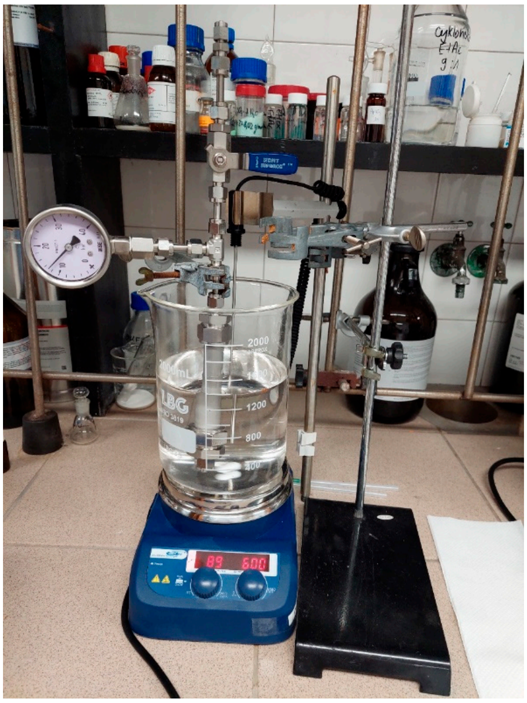

Figure S11. The picture of the reaction set used in the catalytic assays.

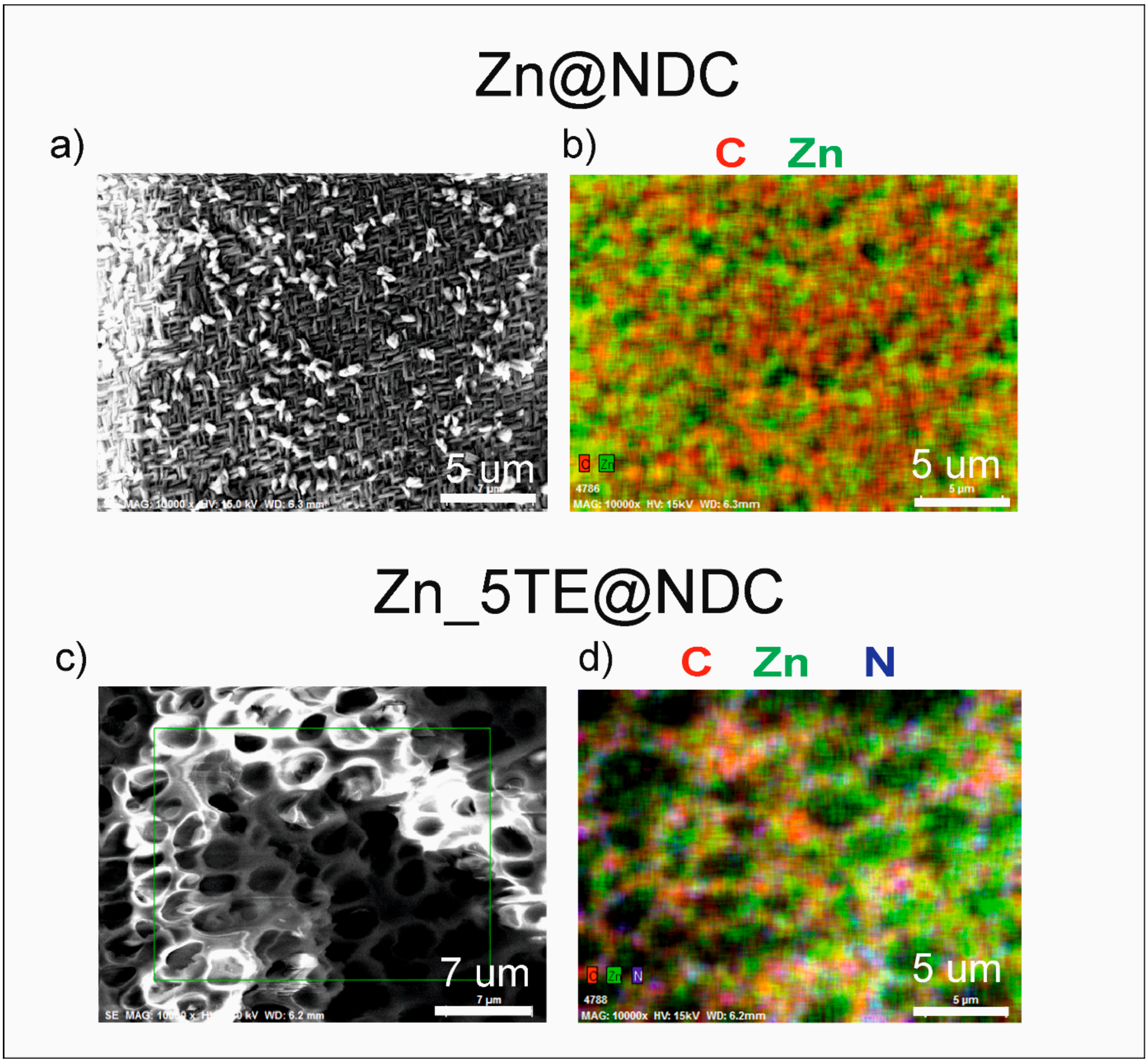

Figure S12. Elemental maps of Zn, C, and N concentrations b) and d) were obtained from SEM-EDS analyses in the sample area shown in a) and c), respectively.

Elemental analysis results for the fabricated nanocatalysts:

Zn_5TE@NDC ($Zn^{2+}(NDC^{2-})_{1,37}(TE)^{+}_{0.75}(TE)_{0.92}$ • 7 H_2O): C: 35.37%, 35.10; H: 2.97%, 2.92 %; N: 18.93%, 18.80%. Calculated: C: 34.38%; H: 4.74%; N: 18.49%.

Zn_5TE_AuNPs@NDC (Zn^{2+} $(NDC^{2-})_{1,18}$ $(TE)^{+}_{0.35}$ $(TE)_{0.78}$ • 0.01 Au • 0.16 S • 2 H_2O):

C: 39.92%, 40.10 %; H: 3.71%, 3.68%; N: 17.54%, 17.33%; S: 1.12%, 1.13%. Calculated: C: 40.09%, H: 3.67%; N: 17.28%; S: 1.12%

Zn_5TE_AuNCs@NDC **(Zn^{2+} $(NDC^{2-})_{0.58}$ $(OH)^{-}_{0.82}$ $(TE)_{0.78}$ • 0.03 Au • 0.15 S):**

C: 35.41%, 35.40%; H: 3.60%, 3.63%; N: 20.59%, 20.71%; S: 1.84%, 1.86%. Calculated: C: 32.8%; H: 2.74%; N: 19.24%; S: 1.69%.

^{1}H NMR for post-reaction mixtures obtained as a result of the synthesis of given carbonates (after TBAB separation via adsorption on silica gel)

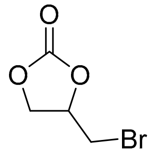

^{1}H NMR (400 MHz, Chloroform-d) δ [ppm] = 4.95 (dq, J = 8.1, 5.5 Hz, 1H), 4.60 (dd, J = 8.9, 8.2 Hz, 1H), 4.36 (dd, J = 8.9, 5.9 Hz, 1H), 3.59 (d, J = 5.2 Hz, 2H).

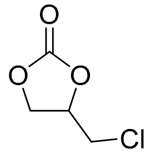

^{1}H NMR (400 MHz, Chloroform-d) δ [ppm] = 5.02 – 4.92 (m, 1H), 4.60 (t, J = 8.5 Hz, 1H), 4.42 (dd, J = 8.9, 5.7 Hz, 1H), 3.84 – 3.69 (m, 2H).

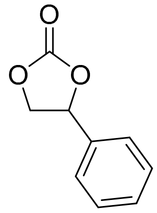

^{1}H NMR (400 MHz, Chloroform-d) δ [ppm] = 7.45 – 7.40 (m, 3H), 7.38 – 7.34 (m, 2H), 5.67 (t, J = 8.0 Hz, 1H), 4.84 – 4.74 (m, 1H), 4.33 (dd, J = 8.7, 7.8 Hz, 1H).

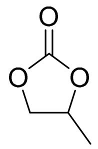

^{1}H NMR (400 MHz, Chloroform-d) δ [ppm] = 4.86 (tq, J = 7.4, 6.2 Hz, 1H), 4.56 (dd, J = 8.4, 7.7 Hz, 1H), 4.03 (dd, J = 8.4, 7.2 Hz, 1H), 1.50 (d, J = 6.2 Hz, 3H).

The spectroscopic data are in agreement with those reported in the literature.[1]

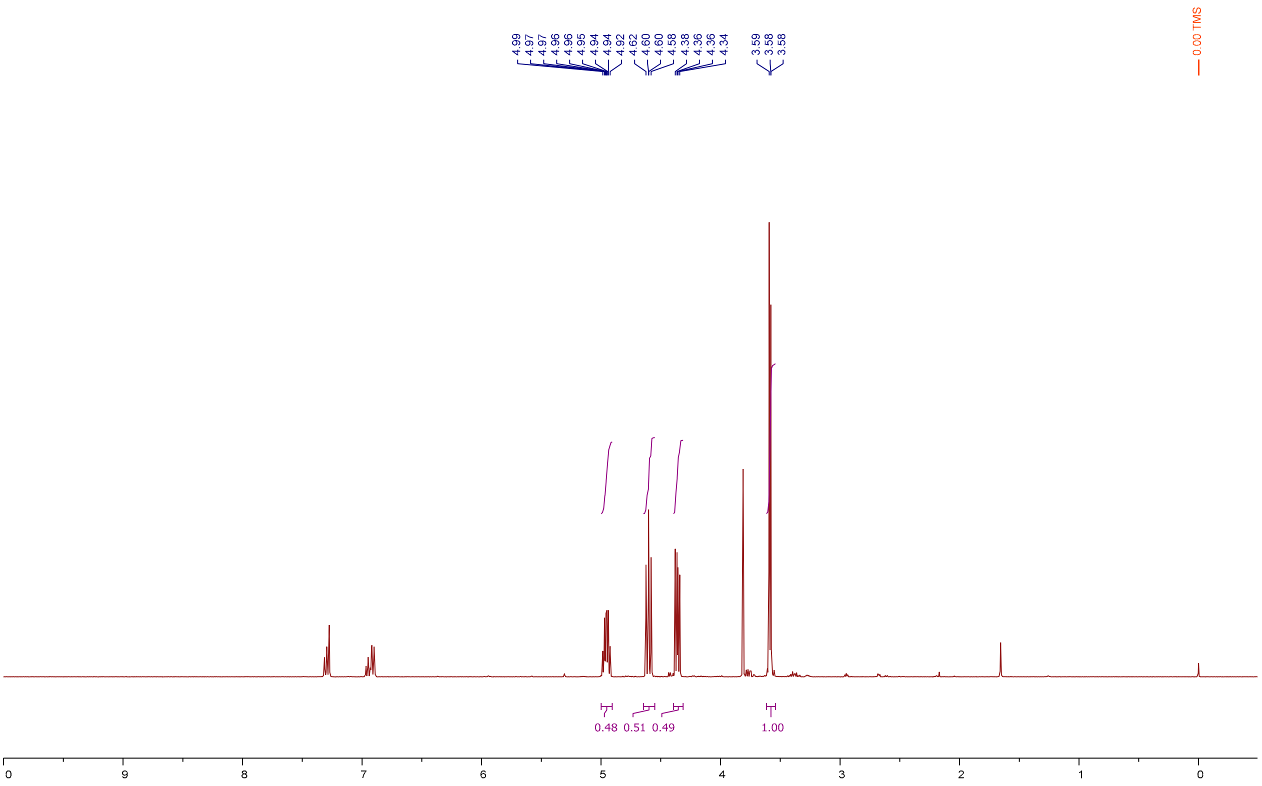

Figure S13. ¹H NMR (400 MHz, Chloroform-d) spectrum of 4-(Bromomethyl)-1,3-dioxolan-2-one with anisole (1:0.167 mol/mol).

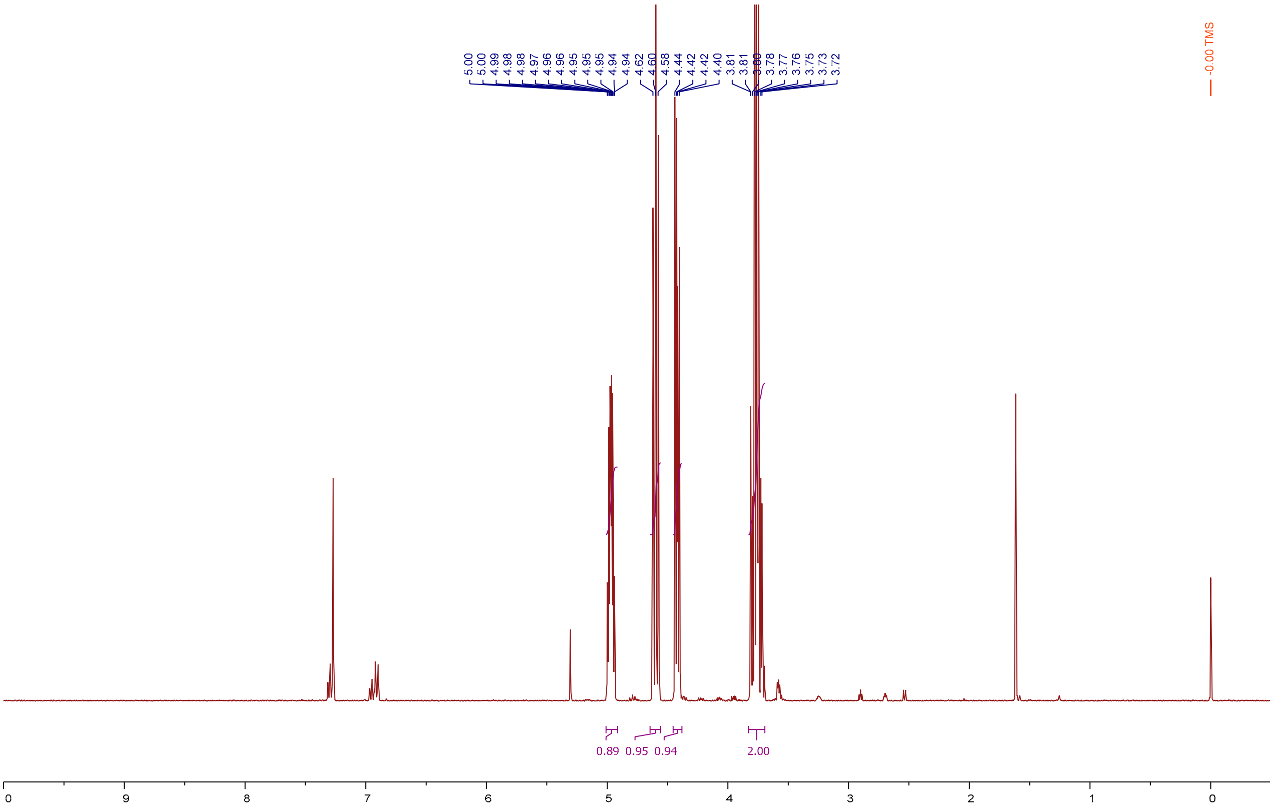

Figure S14. ¹H NMR (400 MHz, Chloroform-d) spectrum of 4-(Chloromethyl)-1,3-dioxolan-2-one with anisole (1:0.141 mol/mol).

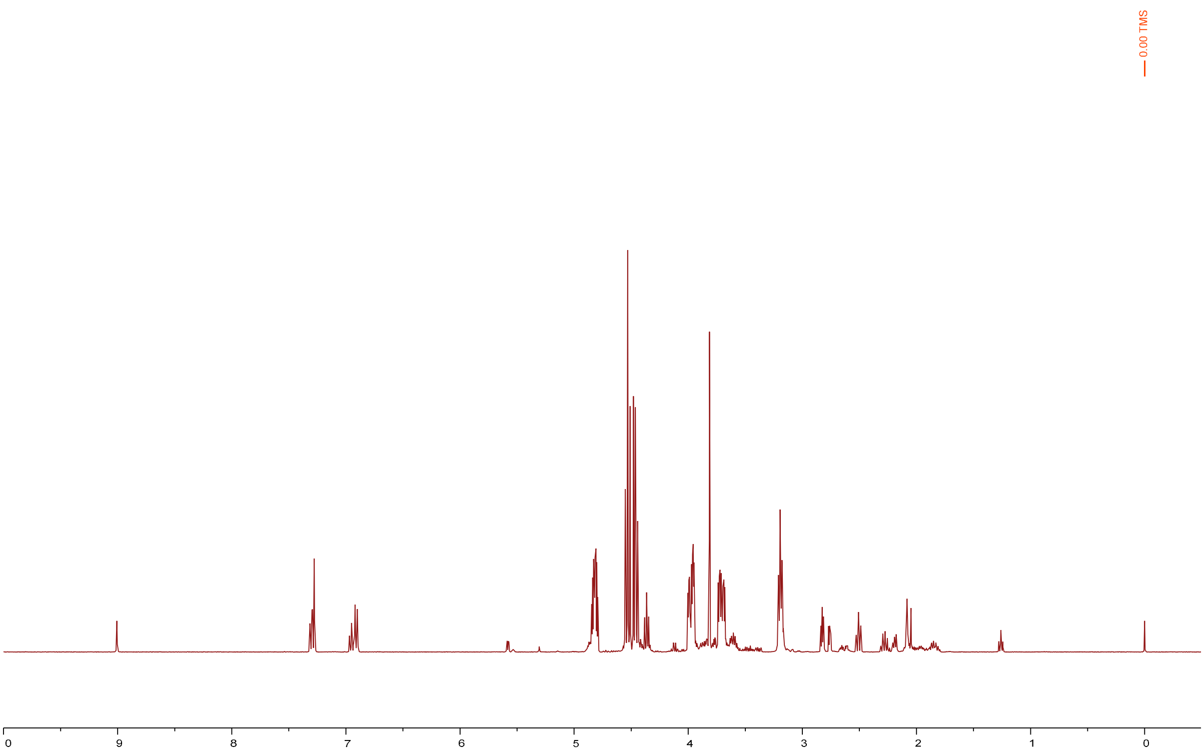

Figure S15. ^{1}H NMR (400 MHz, Chloroform-d) spectrum of 4-(Hydroxymethyl)-1,3-dioxolan-2-one with anisole (1:0.145 mol/mol).

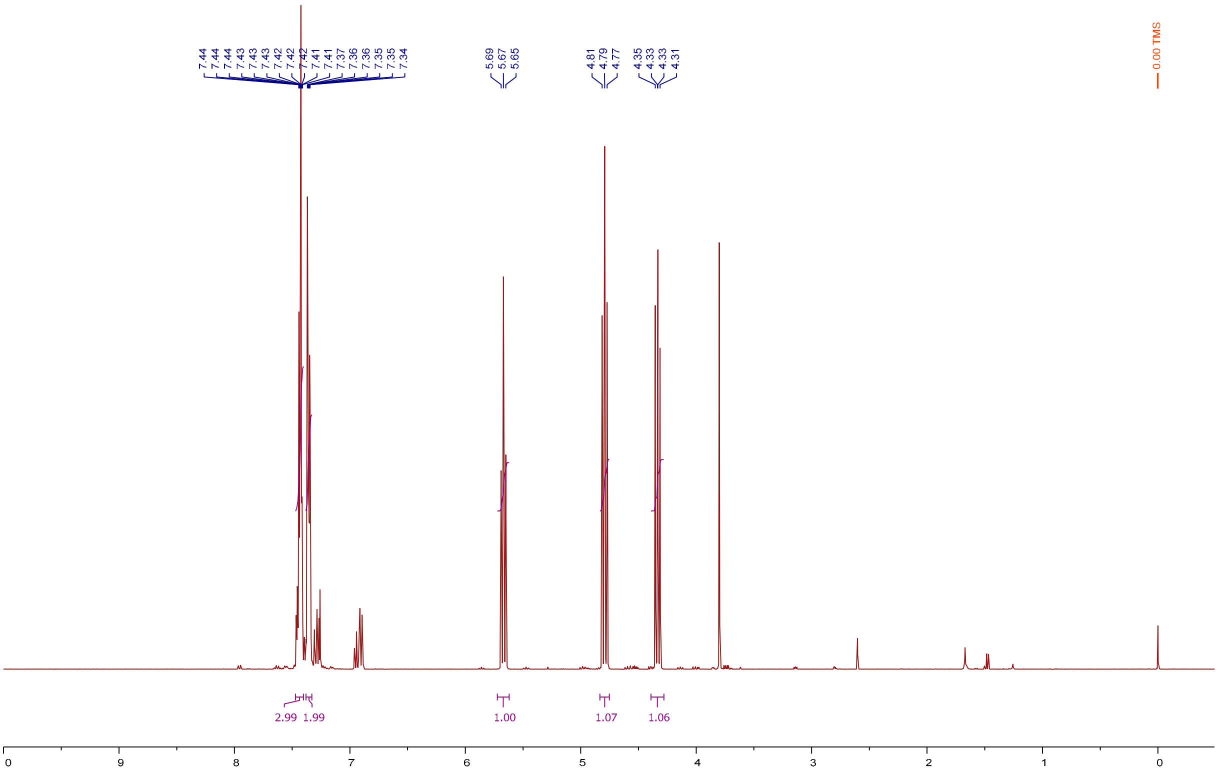

Figure S16. ^{1}H NMR (400 MHz, Chloroform-d) spectrum of 4-Phenyl-1,3-dioxolan-2-one with anisole (1:0.119 mol/mol).

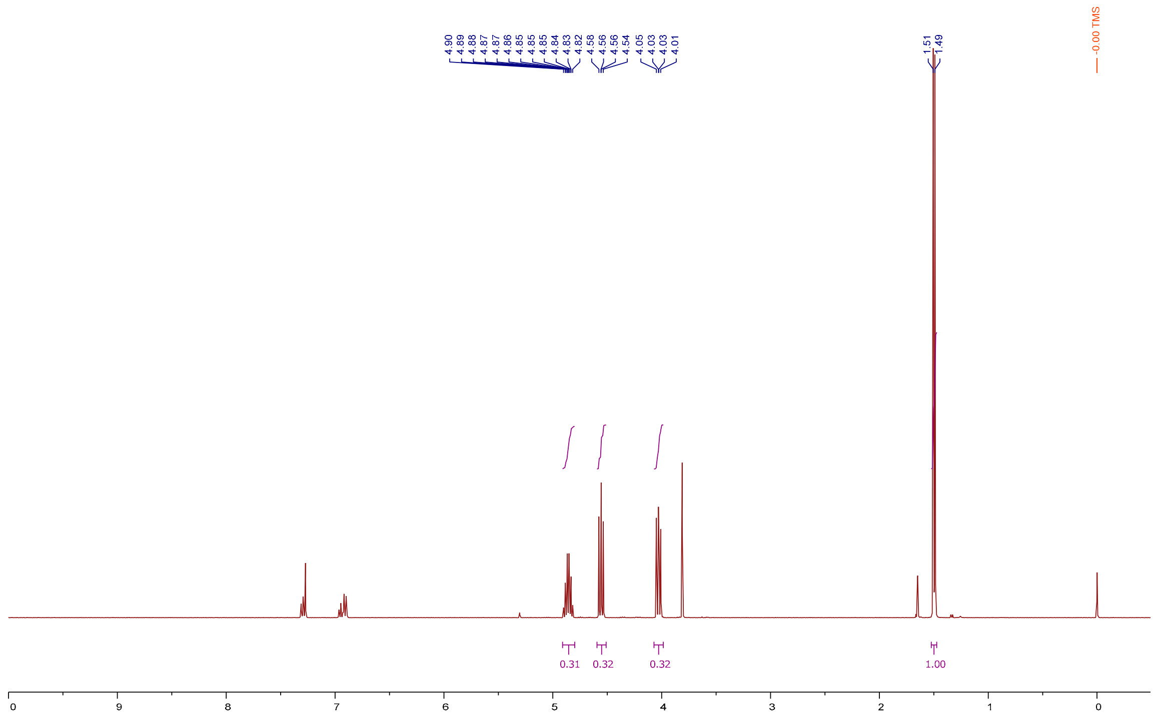

Figure S17. ^{1}H NMR (400 MHz, Chloroform-d) spectrum of 4-Methyl-1,3-dioxolan-2-one with anisole (1:0.138 mol/mol).

Table S1. Comparison of Zn_5TE_AuNCs@NDC with other reported CP systems for the cycloaddition of CO_2 to styrene oxide (SO), leading to styrene carbonate (SC).

Catalyst	Co-catalyst (% mol)	Temperature [°C]	CO_2 pressure [bar]	Time [h]	Solvent	SO Conversion/ SC Yield [%]	Ref.
Zr-(I^-)MeIM-UiO-66	-	120	1	24	-	46	[2]
Cr-MIL-101	TBAB (2%)	25	8	24	-	49/33	[3]
Zn-MOF-5	TBAB (2.5%)	50	60	15	-	n.r./92	[3]
Co-MOF-74	-	100	20	4	Ph-Cl	96/95	[4]
Zr-UiO-66-OH	TBAI (2%)	140	10	2	-	96/n.r.	[5]
Al-MIL-101-NH_2	TBAB (0.14%)	120	18	6	-	95/94	[6]
Y-gea-MOF-1	TBAB (0.15%)	120	20	6		85/n.r.	[6]
Al4@NDC	TBAB (2%)	90	10	22	CH_2Cl_2	28/27	[7]
Al4Au@NDC	TBAB (2%)	90	10	22	CH_2Cl_2	24/23	[7]
Zn_5TE_AuNCs@NDC	TBAB (1.2%)	80	15	24	-	100/94	This work

n.r. – not reported, TBAB – tetrabutylammonium bromide, TBAI – tetrabutylammonium iodide

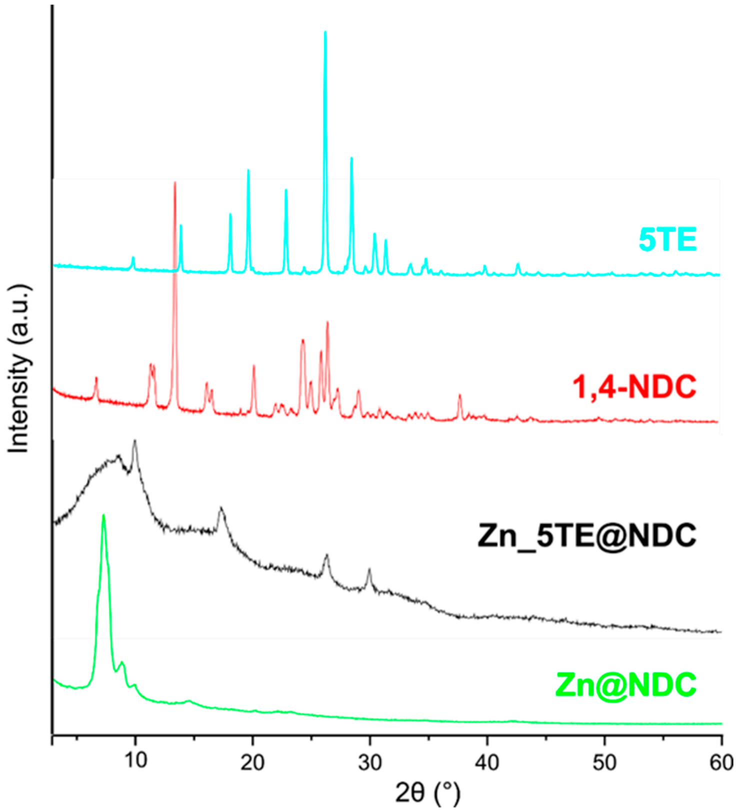

Figure S18. *Powder X-ray diffraction (PXRD) patterns of the CP building blocks – 5-aminotetrazole (5TE, light blue), 1,4-naphtalenedicarboxylic acid (1,4-NDC, red) and CPs - Zn@NDC – green, and Zn_5TE@NDC – black.*

Table S2
SEM-EDS analysis results for Zn_5TE_AuNCs@NDC after its use in the 5th catalytic assay.

Spectrum: Acquisition

Element	Series	unn. C [wt.%]	norm. C [wt.%]	Atom. C [at.%]	Error (1 Sigma) [wt.%]
Carbon	K-series	41.02	43.05	55.53	5.31
Nitrogen	K-series	15.63	16.40	18.14	2.79
Oxygen	K-series	21.91	22.99	22.26	3.21
Sulfur	K-series	1.13	1.18	0.57	0.07
Chlorine	K-series	0.69	0.73	0.32	0.06
Zinc	K-series	11.74	12.32	2.92	0.50
Gold	M-series	3.19	3.34	0.26	0.16
	Total:	95.30	100.00	100.00	

References

[1] H. Phan, P. de la Cruz-Sánchez, M. J. Cabrera-Afonso, B. Martín-Matute, *Green Chem.* **2025**, *27*, 2439-2448.

[2] J. Liang, R.-P. Chen, X.-Y. Wang, T.-T. Liu, X.-S. Wang, Y.-B. Huang, R. Cao, *Chemical science* **2017**, *8*, 1570-1575.

[3] J. Song, Z. Zhang, S. Hu, T. Wu, T. Jiang, B. Han, *Green Chem.* **2009**, *11*, 1031-1036.

[4] H.-Y. Cho, D.-A. Yang, J. Kim, S.-Y. Jeong, W.-S. Ahn, *Catal. Today* **2012**, *185*, 35-40.

[5] J. Noh, Y. Kim, H. Park, J. Lee, M. Yoon, M. H. Park, Y. Kim, M. Kim, *Journal of Industrial and Engineering Chemistry* **2018**, *64*, 478-483.

[6] S. Senthilkumar, M. S. Maru, R. S. Somani, H. C. Bajaj, S. Neogi, *Dalton Transactions* **2018**, *47*, 418-428.

[7] G. Kopacka, K. Wasiluk, P. W. Majewski, M. Kopyt, P. Kwiatkowski, E. Megiel, *International Journal of Molecular Sciences* **2024**, *25*, 1020.
